# Wnt signalling tunes neurotransmitter release by directly targeting Synaptotagmin-1

**DOI:** 10.1038/ncomms9302

**Published:** 2015-09-24

**Authors:** Lorenza Ciani, Aude Marzo, Kieran Boyle, Eleanna Stamatakou, Douglas M. Lopes, Derek Anane, Faye McLeod, Silvana B. Rosso, Alasdair Gibb, Patricia C. Salinas

**Affiliations:** 1 Department of Cell and Developmental Biology, University College London, London WC1E 6BT, UK; 2 Department of Neuroscience, Physiology and Pharmacology, University College London, London WC1E 6BT, UK

## Abstract

The functional assembly of the synaptic release machinery is well understood; however, how signalling factors modulate this process remains unknown. Recent studies suggest that Wnts play a role in presynaptic function. To examine the mechanisms involved, we investigated the interaction of release machinery proteins with Dishevelled-1 (Dvl1), a scaffold protein that determines the cellular locale of Wnt action. Here we show that Dvl1 directly interacts with Synaptotagmin-1 (Syt-1) and indirectly with the SNARE proteins SNAP25 and Syntaxin (Stx-1). Importantly, the interaction of Dvl1 with Syt-1, which is regulated by Wnts, modulates neurotransmitter release. Moreover, presynaptic terminals from Wnt signalling-deficient mice exhibit reduced release probability and are unable to sustain high-frequency release. Consistently, the readily releasable pool size and formation of SNARE complexes are reduced. Our studies demonstrate that Wnt signalling tunes neurotransmitter release and identify Syt-1 as a target for modulation by secreted signalling proteins.

## Abstract (web summary)

The mechanisms by which signalling proteins dynamically modulate neurotransmitter release remain poorly understood. Here, Ciani *et al*. show Wnt signalling influences vesicle pool availability in an activity-dependent manner via direct interactions with Dishevelled-1 and the synaptic vesicle calcium sensor Synaptotagmin-1.

## Graphical abstract

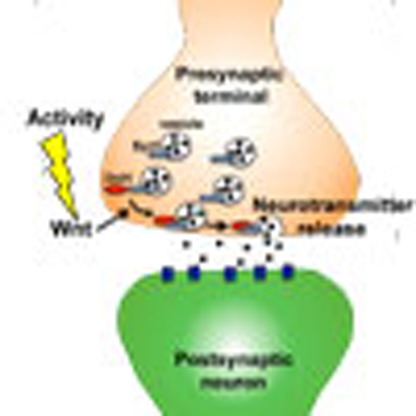

The arrival of an action potential (AP) at the presynaptic terminal induces the entry of Ca^2+^ through voltage-gated calcium channels, triggering synaptic vesicle exocytosis and the release of neurotransmitters^1^. Synaptic vesicles go through a series of well-characterized steps that culminate with the formation of a readily releasable pool (RRP) of docked vesicles, which can rapidly fuse with the plasma membrane upon Ca^2+^ influx. Although significant progress has been made in elucidating the molecular steps leading to synaptic vesicle docking, fusion, release and retrieval^1^,^2^,^3^,^4^,^5^, little is known about the mechanisms by which extracellular signalling proteins modulate neurotransmitter release.

SNAREs (soluble N-ethylmaleimide-sensitive fusion proteins) are the core molecules that control synaptic vesicle release competence and exocytosis. SNAREs form a complex that includes the vesicular protein Synaptobrevin/VAMP2 (v-SNARE) and the plasma membrane proteins Stx-1 and SNAP25 (t-SNAREs)^6^,^7^,^8^,^9^. A rise in Ca^2+^ concentration brings synaptic vesicles into close proximity with the plasma membrane through the interaction between v-SNAREs and t-SNAREs^6^,^7^,^8^. Binding of Ca^2+^ to Synaptotagmin-1 (Syt-1), a key synaptic vesicle protein and a calcium sensor, results in a conformational change that facilitates rapid fusion of synaptic vesicles with the plasma membrane^10^,^11^,^12^,^13^,^14^,^15^. In addition, Syt-1 has been shown to regulate vesicle docking in chromaffin cells^16^ and at central synapses^17^. Ca^2+^ entry is the primary trigger initiating neurotransmitter release. However, this process can also be modulated by extracellular signals to allow synapses to adapt to changes in demands.

Secreted proteins that signal at the synapse could act as tonic modulators of neurotransmitter release. Indeed, a well-known regulator of neurotransmitter release is brain-derived neurotrophic factor (BDNF). At CA1 synapses, BDNF increases the number of docked vesicles and quantal neurotransmitter release^18^,^19^,^20^. Conversely, loss of function of BDNF results in fewer docked vesicles and synaptic depression upon high-frequency stimulation (HFS)^21^. However, the mechanisms involved remain elusive. In addition to BDNF, Wnts are emerging as key signalling molecules that regulate synapse formation and synaptic transmission^22^,^23^,^24^. Gain and loss of function studies have demonstrated that Wnts directly signal to the axon to promote the assembly of presynaptic release sites during synaptogenesis^25^. Analyses of miniature currents in the cerebellum of Wnt-deficient mice^25^ and in hippocampal neurons upon application of exogenous Wnts^26^ have suggested a possible role for Wnts in neurotransmitter release. However, key questions remain unanswered: does Wnt signalling modulate transmitter release *in vivo*? What are the mechanisms underpinning this modulation?

Here we report that Wnt7a, acting through Dishevelled-1 (Dvl1), a scaffold protein and integrator of Wnt signalling that determines the cellular locale of Wnt action, directly modulates core proteins of the presynaptic release machinery at excitatory synapses of the hippocampus. We demonstrate that Dvl1 interacts indirectly with the t-SNAREs SNAP25 and Stx-1, and directly with the calcium sensor Syt-1 at synapses. Although overexpression studies using cell lines have reported a possible interaction between Dvl1 and Syt-1 (ref. 27), the physiological significance of this interaction has remained elusive. Here we show that endogenous Dvl1 and Syt-1 indeed interact at central synapses. Moreover, we demonstrate that this interaction is increased by Wnts and decreased by secreted Wnt antagonists. Consistently, *Wnt7a; Dvl1* double-mutant mice exhibit defects in the formation of the SNARE complex, a decreased number of synaptic vesicles proximal to release sites and a decreased RRP size. These mutants also manifest defects in neurotransmitter release probability and quantal content at excitatory hippocampal synapses. Importantly, these defects in neurotransmitter release can be phenocopied by presynaptically interfering with the interaction between Dvl1 and Syt-1. Our findings outline a mechanism whereby during synaptic adaptation, extracellular signals such as Wnts modulate neurotransmitter release by targeting the calcium sensor Syt-1. We also show that Wnts contribute to activity-mediated modulation of neurotransmitter release suggesting that Wnt factors play a role in synaptic adaptation.

## Results

### Wnts regulate neurotransmitter release in the hippocampus

Previous studies have shown that exogenous Wnts regulate presynaptic function in hippocampal neurons^26^. However, the *in vivo* requirement for Wnt signalling in neurotransmitter release has not been investigated. To address this issue, we examined Wnt-deficient mice lacking both Wnt7a and Dvl1 (*Wnt7a; Dvl1* knock-out (KO)), as these mice exhibit a stronger phenotype than *Wnt7a* or *Dvl1* single mutants^25^,^28^. We have previously shown that excitatory synapse formation is impaired in the CA3 region of *Wnt7a; Dvl1* KO mice; dendritic spine size and density and the frequency and amplitude of miniature excitatory postsynaptic currents (mEPSCs) are also reduced at CA3 pyramidal cells^29^. However, spine density, mEPSCs and miniature inhibitory postsynaptic currents (mIPSCs) are unaffected in CA1 pyramidal neurons in this mutant^29^ (Supplementary Fig.1). We therefore examined the contribution of Wnt signalling to neurotransmitter release at the Schaffer collateral (SC)-CA1 synapse of the *Wnt7a; Dvl1* mutant mouse, to minimize any confounding effects due to synapse formation defects.

**Figure 1:**
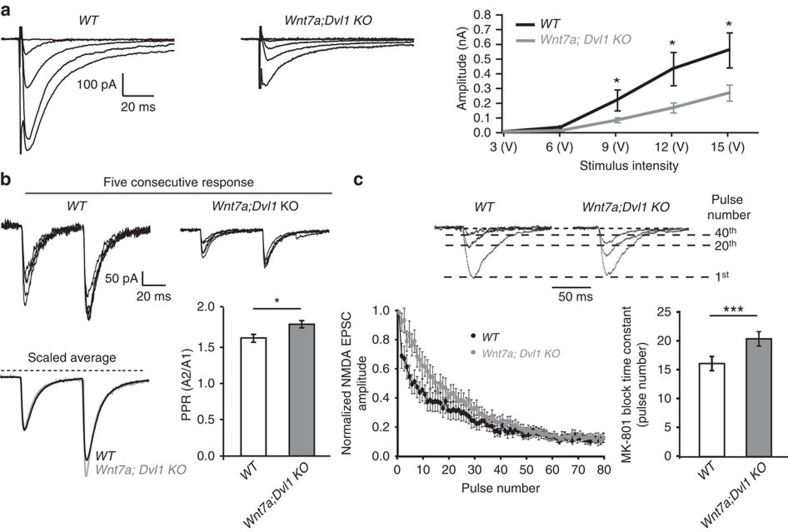
Wnt7a-Dvl1 signalling regulates release probability at glutamatergic synapses in the hippocampus. (**a**) Input–output relationship of evoked EPSCs at SC-CA1 synapses of P21 WT and *Wnt7a; Dvl1* KO hippocampal slices. Traces show responses of representative cells at increasing stimulus voltages (average of three to five responses for each stimulus strength). Graph shows EPSC mean amplitude (mean±s.e.m.) at each stimulus voltage from all cells. Higher stimulus intensities reveal a significant reduction in EPSC amplitude in *Wnt7a; Dvl1* KO mice (*n*=10 cells from four WT animals, 14 cells from three *Wnt7a; Dvl1* KO animals; *P<0.05, unpaired Student's *t*-test). (**b**) PPR of EPSCs at SC-CA1 synapses of P21 WT and *Wnt7a; Dvl1* KO hippocampal slices. Traces show five consecutive overlaid responses of representative cells, and the average response from these cells scaled to the first response. Graph shows the mean PPR (±s.e.m.) from all cells. A significant increase in PPR (corresponding to a decrease in *P*_r_) is observed in *Wnt7a; Dvl1* KO mice (*n*=15 cells from four WT animals and 15 cells from five *Wnt7a; Dvl1* KO animals; *P<0.05, unpaired Student's *t*-test). (**c**) Top: representative traces of three pulse-evoked NMDA EPSCs for the WT and the *Wnt7a; Dvl1* KO mice in presence of MK-801 (30 μM; pulses number 1, 20 and 40). Left panel: normalized peak amplitude of synaptic NMDA currents is plotted versus stimulus trial during MK-801 bath application. Right panel: *Wnt7a; Dvl1* KO animals show a significant increase in the block time constant compared with controls (*n*=11 cells from five WT animals and five cells from four *Wnt7a; Dvl1* KO animals; ***P<0.001, unpaired Student's *t* test).

To investigate defects in evoked glutamatergic transmission, we used acute hippocampal slices from Wnt signalling-deficient mice. Input–output measurements demonstrate that basal evoked transmission is defective in the *Wnt7a; Dvl1* mutant, with a significant reduction in EPSC amplitude at higher stimulus intensities compared with wild-type (WT) (Fig. 1a). A number of factors could contribute to this reduction in basal transmission, including reduced postsynaptic α-amino-3-hydroxy-5-methyl-4-isoxazolepropionic acid receptor (AMPA-R) content, reduced axonal excitability and presynaptic defects in the neurotransmitter release machinery. To specifically test for a presynaptic defect, we evoked EPSCs in quick succession at SC-CA1 synapses and examined the paired-pulse ratio (PPR), which is known to depend on presynaptic short-term plasticity mechanisms reflecting release probability (*P*_r_)^30^,^31^,^32^. We found that PPR is significantly increased in the *Wnt7a; Dvl1* KO when compared with WT (Fig. 1b), suggesting that *P*_r_ is reduced at glutamatergic synapses of Wnt-deficient mice. A complementary test to monitor *P*_r_ consists of measuring the rate of blockade of N-methyl-D-aspartate receptor (NMDA-R)-mediated currents by the use-dependent NMDA-R blocker MK-801 (refs 33, 34). As expected, bath administration of MK-801 during AMPA-R and γ-aminobutyric acid receptor (GABA-R) blockade induced a progressive block of NMDA-R-mediated EPSCs (Fig. 1c) that followed an exponential time course. We found that the rate of decline of NMDA-R-mediated EPSCs induced by MK-801 is significantly slower in *Wnt7a; Dvl1 KO* mice compared with WT (Fig. 1c), indicating a marked decrease in glutamatergic *P*_r_ in these mice. In contrast to excitatory synapses, we found that both basal inhibitory transmission and IPSC PPR are unaffected in *Wnt7a; Dvl1 KO* mice (Supplementary Fig. 2a,b). This might be due to a lack of Wnt7a receptors at inhibitory synapses.

Furthermore, when the average quantal content of AMPA-R-mediated EPSCs was calculated from the ratio of EPSC to mEPSC amplitude, the number of quanta released in response to each stimulus is significantly reduced by 45% in the *Wnt7a; Dvl1* KO compared with WT, demonstrating a presynaptic defect (Supplementary Fig. 1e). Interestingly, we observed a more pronounced defect (a decrease of 69% in quantal content) in the mutant when we used a higher frequency (20 Hz), which could mimic global activity during explorative behaviour^35^,^36^,^37^,^38^ (Supplementary Fig. 1f). Together, these findings suggest that endogenous Wnt7a-Dvl1 signalling specifically regulates *P*_r_ at glutamatergic synapses in the CA1 region in an activity-dependent manner.

### Acute changes in Wnt signalling affect neurotransmitter release

**Figure 2:**
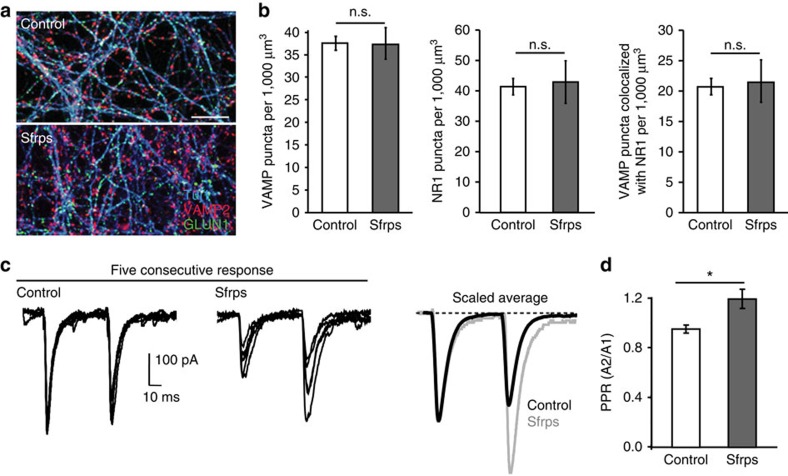
Acute blockade of Wnt signalling lowers release probability at glutamatergic synapses. (**a**) Representative images of mature (21 DIV) hippocampal cultures treated for 3 h with control or a cocktail of Sfrps 1, 2 and 3 and immunostained for the presynaptic marker VAMP2 (red), the postsynaptic marker GluN1 (green) and the cytoskeletal marker Tuj1 (blue). Scale bar, 10 μm. (**b**) Quantification reveals no changes in VAMP2 or GluN1 puncta density (normalized to Tuj1 volume) in response to Sfrps when compared with controls. In addition, the density of synapses (VAMP2 puncta that colocalize with GluN1) is unaffected by Sfrps (8–10 fields from each experiment from three independent experiments. n.s.=nonsignificant, unpaired *t*-test). (**c**) PPR of EPSCs from hippocampal cultures treated with control or a cocktail of Sfrps 1, 2 and 3 for 3 h (*n*=17 cells from 10 cultures for control, 16 cells from 8 cultures for Sfrps). Left-hand and middle traces show five overlaid consecutive traces from representative control or Sfrp-treated cells, respectively. Right-hand traces show the average of all responses from these cells, scaled to the size of the first response. (**d**) The mean PPR of all control and Sfrp-treated cells. An increase in the PPR is observed in neurons exposed to Sfrps. *P<0.05, unpaired Student's *t*-test.

Given that the *Wnt7a; Dvl1* KO mice are constitutively deficient in Wnt signalling, we examined the effect of acute blockade of Wnts using the specific secreted Wnt antagonists secreted frizzled-related proteins 1, 2 and 3 (Sfrps)^39^,^40^,^41^. In mature hippocampal neurons (∼21 days *in vitro* (DIV)), acute exposure to Sfrps did not affect the number of excitatory synapses (Fig. 2a,b) but recapitulates the phenotypes observed in *Wnt7a; Dvl1* KO mice as the EPSC amplitude is reduced at higher stimulus intensities in input–output experiments (Supplementary Fig. 2c) and the EPSC PPR is increased, consistent with a decrease in *P*_r_ (Fig. 2c,d). Again, both basal inhibitory transmission and IPSC PPR are unaffected by Sfrps (Supplementary Fig. 2d,e). Together, these findings demonstrate that deficiency in Wnt signalling decreases *P*_r_ at excitatory synapses without affecting synapse number in mature neurons.

**Figure 3:**
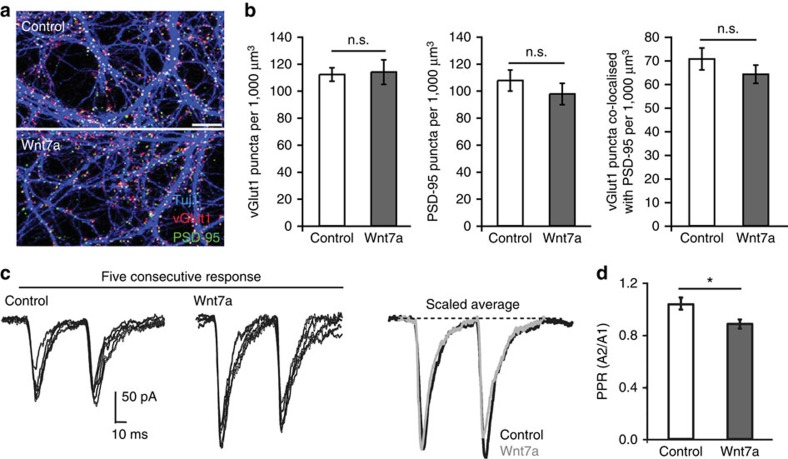
Acute exposure to Wnt7a increases release probability without affecting synapse number in mature hippocampal neurons. (**a**) Representative images of control and Wnt7a-treated (3 h) mature (21 DIV) hippocampal cultures immunostained for the presynaptic marker vGlut1 (red), the postsynaptic marker PSD-95 (green) and the cytoskeletal marker Tuj1 (blue). Scale bar, 10 μm. (**b**) Quantification of vGlut1 or PSD-95 puncta density (normalized to Tuj1 volume) reveals no change in response to Wnt7a compared with control neurons. The density of excitatory synapses (vGlut1 puncta that colocalize with PSD-95) is unaffected by Wnt7a (*n*=8–10 fields for each experiment from three independent experiments. n.s.=nonsignificant, unpaired Student's *t*-test). (**c**) PPR EPSC recordings from hippocampal cultures treated with control or Wnt7a for 3 h (*n*=17 cells from 11 control cultures and 17 cells from 11 Wnt7a-treated cultures). Left-hand and middle traces show five overlaid consecutive traces from representative control or Wnt7a-treated cells, respectively. Right-hand traces show the average of all responses from these cells, scaled to the size of the first response. (**d**) The mean PPR of all control and Wnt7a-treated cells. A decrease in the PPR is observed in cells exposed to Wnt7a. *P<0.05, unpaired Student's *t*-test.

We next examined the effect of gain of function of Wnt signalling on *P*_r_. As we have previously shown that Wnt7a increases the number of excitatory synapses in young neurons^25^,^29^, we first tested whether Wnt7a affects synapse number in mature neurons. Acute Wnt7a treatment did not affect excitatory synapse number (Fig. 3a,b) yet decreased the EPSC PPR (indicating an increase in *P*_r_; Fig. 3c,d). In addition, EPSC amplitude is increased at higher stimulus intensities in input–output experiments on Wnt7a exposure (Supplementary Fig. 2c). Basal inhibitory transmission is unaffected by Wnt7a treatment (Supplementary Fig. 2d). Together, these results strengthen the conclusion that Wnt signalling regulates *P*_r_ at glutamatergic synapses in mature neurons independently of changes in synapse number.

The finding that Wnt7a modulates neurotransmitter release led us to propose that Wnts might contribute to synaptic adaptation. To test this possibility, we examined the impact of Wnt blockade on release probability when neurons are exposed to HFS, a well-known paradigm that increases release probability^32^,^42^,^43^,^44^. For this experiment, hippocampal brain slices were exposed to Sfrps during HFS. PPR was quantified 1 min before and after the HFS. In control conditions, PPR is reduced after HFS consistent with an increase in *P*_r_ (Supplementary Fig. 3a), as described before^32^,^42^,^43^,^44^. In contrast, Sfrps block the effect of HFS on PPR (Supplementary Fig. 3b). Our findings suggest that Wnts regulate neurotransmitter release in an activity-dependent manner and therefore modulate release probability according to demand.

### Wnt signalling regulates synaptic vesicle number and RRP size

**Figure 4:**
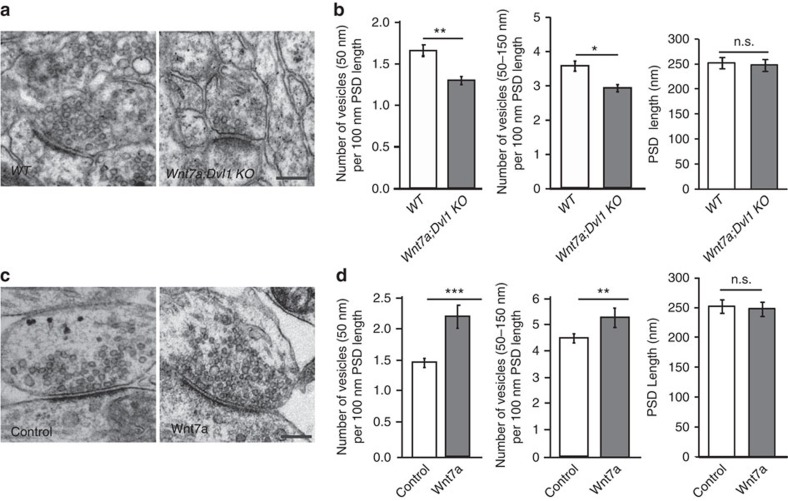
Wnt signalling regulates synaptic vesicle number. (**a**) Representative electron micrographs of a typical asymmetric synapse in the CA1 of the hippocampus of WT and *Wnt7a; Dvl1* KO mice. Scale bar, 200 nm. (**b**) *Wnt7a; Dvl1* KO mice exhibit defects in the number of vesicles within 50 nm from the active zone and within 50–150 nm from the active zone. Vesicle number was normalized to the length of the PSD, which is constant between genotypes. Data are from three WT mice (124 synapses) and three *Wnt7a; Dvl1* KO mice (123 synapses). **P<0.01, *P<0.05 Kruskal–Wallis test. (**c**) Representative electron micrographs of a typical asymmetric synapse in mature hippocampal cultures treated overnight with control or Wnt7a. Scale bar, 200 nm. (**d**) Treatment with Wnt7a increases the number of synaptic vesicles within 50 nm from the active zone and within 50–150 nm from the active zone in mature neurons. Number of vesicles was normalized to the PSD length, which is not affected by Wnt7a. Data are from three independent experiments (Control: 99 synapses; Wnt7a: 116 synapses). **P<0.01, ***P<0.001, Kruskal–Wallis test.

The arrival of an AP at the presynaptic terminal and Ca^2+^ influx leads to the fusion of synaptic vesicles^2^,^6^. We therefore examined whether Wnt signalling deficiency affects the distribution of synaptic vesicles that could contribute to neurotransmitter release defects by performing ultrastructural analyses at the SC-CA1 synapse (Supplementary Fig. 4). Docked vesicles have previously been defined according to different criteria^16^,^45^,^46^,^47^. Following the more strict criterion where docked vesicles are defined as those directly touching the active zone, no difference is observed in the number of these vesicles in the *Wnt7a; Dvl1* KO or in mature neurons exposed to Wnt7a when compared with controls. However, when we quantified the number of vesicles proximal to the plasma membrane (within a distance of 50 nm), we found that the *Wnt7a; Dvl1* KO mice exhibit a reduced number of such vesicles (Fig. 4a,b), whereas acute exposure to Wnt7a elicits the opposite effect on cultured neurons (Fig. 4c,d). Furthermore, the number of vesicles within a region spanning a distance of 50–150 nm from the plasma membrane is also decreased in the *Wnt7a; Dvl1* KO. Conversely, Wnt7a increases the number of vesicles (Fig. 4a–d). These findings demonstrate that Wnt signalling regulates the number of synaptic vesicles at the presynaptic terminal.

**Figure 5:**
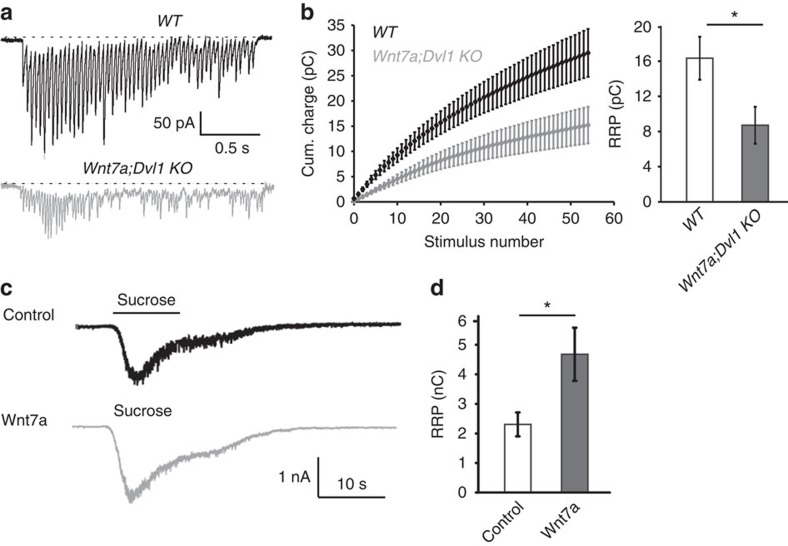
Wnt signalling regulates the RRP size. (**a**) Representative traces of EPSCs evoked by electrical stimulation (20 Hz for 2.5–3 s per train) recorded from WT and *Wnt7a; Dvl1* KO CA1 cells. Note the reduced amplitude of the responses in the *Wnt7a; Dvl1* KO cell compared with a WT neuron. (**b**) Average cumulative charge versus stimulus number for all cells from each genotype (left) and RRP size (in term of total charge) estimated with first-order correction for vesicle refilling during stimulus trains in each cell (right, WT=16.3±2.4 pC; *Wnt7a; Dvl1* KO=8.7±2.1 pC, *n*=13 cells from three animals for WT, 12 cells from three animals from *Wnt7a; Dvl1* KO). *P<0.05; unpaired Student's *t*-test. (**c**) Representative responses evoked by hypertonic sucrose application from neurons exposed to control (black) or Wnt7a (grey). (**d**) Wnt7a increases the mean charge transfer-transient phase of sucrose-induced responses, corresponding to the RRP size (apparent RRP size estimate) in hippocampal mature neurons (Control: 29 cells; Wnt7a: 18 cells). *P<0.05; unpaired Student's *t*-test.

Several studies have demonstrated a strong correlation between *P*_r_ and the size of the RRP, which contains vesicles that are available for release immediately on stimulation^5^,^19^,^30^,^48^,^49^,^50^,^51^,^52^,^53^. To further define the role of Wnt signalling in neurotransmitter release, we analysed the size of the RRP at the SC-CA1 synapse. Using brief trains of HFS^51^,^54^, we observed a significant reduction in the size of the RRP in acute slices as analysed with first-order correction for vesicle recycling^55^ (Fig. 5a,b). These analyses revealed that the fusion efficiency (*f*e) and the refilling rate constant (*α*) are similar in both genotypes (Supplementary Fig. 5a–c). Fitting a simple model of short-term facilitation of transmitter release to the EPSC quantal content during trains of stimulation demonstrated that a reduced RRP (46%) can account for the general decrease in *P*_r_ in *Wnt7a; Dvl1* KO mice (Supplementary Fig. 5d). Furthermore, we used hypertonic sucrose to measure the size of the RRP in mature hippocampal neurons. We found that Sfrps decrease whereas Wnt7a increases the RRP size when compared with control neurons (Supplementary Fig. 5e and f and Fig. 5c,d). Taken together, these results demonstrate that Wnt signalling regulates the number of synaptic vesicles close to the active zone and the size of the RRP, which could account for the changes in *P*_r_.

### Wnt signalling deficiency compromises SNARE assembly

**Figure 6:**
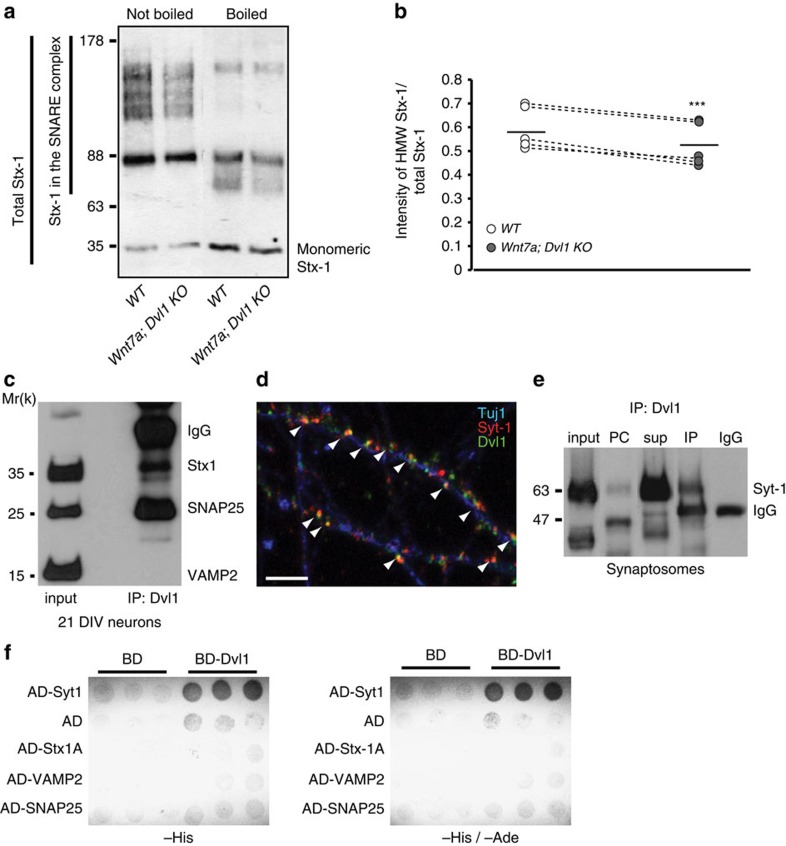
Wnt signalling regulates SNARE complex formation and Dvl1 interacts with proteins of the release machinery. (**a**) Representative SNARE preparations from two hippocampi of *Wnt7a; Dvl1* KO and WT mice. The two lines on the left represent the region of Stx-1-containing complexes and the total amount of Stx-1, respectively. (**b**) Quantification shows a decrease in the Stx-1 complex in the *Wnt7a; Dvl1* KO. *n*=5 WT and five mutant mice from three different experiments. ***P<0.001, two-way ANOVA with blocking. (**c**) Representative immunoprecipitation samples of SNARE proteins from mature hippocampal neurons show that Dvl1 interacts with SNAP25 and Syt-1, but not with VAMP2. Full-length blot provided in Supplementary Fig 7a. (**d**) In mature hippocampal neurons, Dvl1 (green) colocalizes with Syt-1 (red) along the axon (Tuj1, blue), see arrowhead. Scale bar, 5 μm. (**e**) Immunoprecipitation experiments show that Dvl1 interacts with Syt-1 in synaptosomes prepared from brain homogenate. Input, Input; PC, pre-clear; sup, supernatant; IP, immunoprecipitate and IgG, antibody. Full-length blot provided in Supplementary Fig 7b. (**f**) Dvl1 was fused to the GAL4 DNA-BD and tested for interactions against SNARE proteins fused to the GAL4 AD. Growth on −His and −His/−Ade media shows that Dvl1 has a strong and direct interaction with Syt-1, but not with empty vector (AD) or with Stx-1, VAMP2 or SNAP25.

The fusion of vesicles at the presynaptic terminal is highly dependent on the assembly of the SNARE complex^2^,^6^,^7^,^8^. Our finding that *Wnt7a; Dvl1* mutant mice exhibit defects in the number of synaptic vesicles and the size of the RRP prompted us to investigate whether Wnt signalling affects the assembly of the SNARE complex. It is well documented that SNARE proteins form a high-affinity four-helical bundle that is resistant to disruption by the denaturing detergent SDS, but which can be dissociated if boiled in the presence of SDS^56^,^57^. To examine changes in SNARE complex formation, we analysed the proportion of Stx-1 in the SDS-resistant SNARE complex over the total level of Stx-1 in the unboiled preparation from hippocampal lysates. Using this method, we found a significant reduction in the level of the t-SNARE Stx-1 in the SDS-resistant unboiled complex in the Wnt-deficient mutant (Fig. 6a,b), suggesting that the assembly of the SNARE complex is compromised when Wnt signalling is defective in the hippocampus.

### Dvl1 directly interacts with the calcium sensor Syt-1

Dvl1 is a scaffold protein and a key integrator of the Wnt signalling cascade that localizes to specific cellular compartments to regulate protein–protein interactions and/or enzyme activity in a Wnt-dependent manner^58^. Our previous studies have demonstrated that Dvl1 is present at presynaptic sites in central synapses^25^. On the basis of these findings, we hypothesized that Wnt signalling could regulate neurotransmitter release by modulating the interaction of Dvl1 with components of the SNARE complex. We therefore performed immunoprecipitation experiments to screen for proteins of the SNARE complex that interact with Dvl1. We found that endogenous Dvl1 interacts with the t-SNARE proteins Stx-1 and SNAP25 in mature hippocampal neurons (Fig. 6c). Surprisingly, the v-SNARE protein VAMP2 is not detected in a complex with Dvl1 (Fig. 6c).

We next investigated the interaction of Dvl1 with Syt-1, a core protein for neurotransmitter release^10^,^11^,^14^,^15^. Colocalization between Dvl1 and Syt-1 along the axon of mature neurons suggests that these proteins could indeed interact at presynaptic sites (Fig. 6d). Importantly, immunoprecipitation experiments using synaptosomal fraction from the adult brain demonstrate that endogenous Dvl1 and Syt-1 proteins co-precipitate (Fig. 6e). Interestingly, Dvl1 also interacts with the cytomatrix protein CASK, but not with Munc-18 or Complexin, two presynaptic proteins involved in Ca^2+^-mediated exocytosis (Table 1). Unfortunately, because of incompatibility of the available antibodies, we were unable to examine the possible interaction between Dvl1 and RIM, a key protein involved in the priming of synaptic vesicles^2^. Together, these findings demonstrate that Dvl1 specifically interacts with Stx-1, SNAP25, Syt-1 and CASK, important components of the release machinery.

To investigate whether the interaction of Dvl1 with Syt-1 and the t-SNARE proteins is direct, we performed yeast two-hybrid assays using full-length Dvl1 as bait and Syt-1, Stx-1, VAMP2 and SNAP25 as prey (Fig. 6f). We found that Dvl1 interacts directly with Syt-1, as revealed by −Histidine nutrition selection (Fig. 6f; left panel). Importantly, this interaction is strong, since we could also detect it under more stringent selection (−Histidine/−Adenine, Fig. 6f; right panel). However, we did not detect any direct interaction between Dvl1 and Stx-1, SNAP25 or VAMP2. Together, these results demonstrate that Dvl1 directly binds to Syt-1, whereas the interaction with the t-SNARE proteins, Stx-1 and SNAP25, is indirect.

### Wnts modulate the interaction between Dvl1 and Syt-1

**Figure 7:**
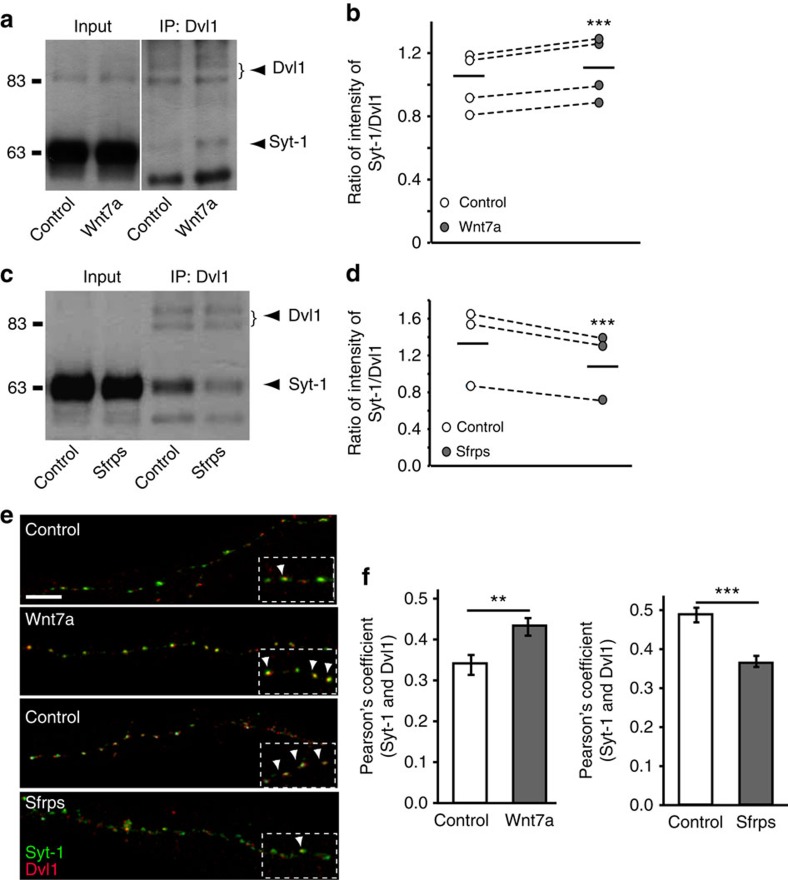
Wnts modulate the interaction between Dvl1 and Syt-1. (**a**) Hippocampal neurons were exposed to Wnt7a, and the interaction of endogenous Dvl1 and Syt-1 was examined using immunoprecipitation. (**b**) Quantification of the intensity of Syt-1 normalized to Dvl1 (*n*=4 independent experiments). ***P<0.001, two-way ANOVA with blocking. Full-length blot provided in Supplementary Fig 7c. (**c**) Immunoprecipitation of mature hippocampal neurons exposed to Sfrps. (**d**) Quantification of the intensity of Syt-1 was normalized to Dvl1 (*n*=3 independent experiments). Full-length blot provided in Supplementary Fig 7d. ***P<0.001, two-way ANOVA with blocking. (**e**) Axons of isolated hippocampal neurons exposed to Wnt7a or Sfrps and stained for endogenous Syt-1 and Dvl1. Arrowheads highlight colocalization between Syt-1 and Dvl1. Fourteen and twenty-one DIV cultures were exposed to Wnt7a and Sfrps, respectively, to maximize the effect of gain or loss of function of Wnts. Scale bar, 5 μm. (**f**) Quantification of colocalization using Pearson's correlation along the axon of Wnt7a- or Sfrps-treated neurons (*n*=3 independent experiments; 10 axons per condition per experiment were used). **P<0.01, ***P<0.001 unpaired Student's *t*-test.

Activation of the Wnt pathway regulates the localization of Dvl to specific cellular locale and its interaction with distinct intracellular molecules^58^. We therefore decided to examine whether Wnts modulate the binding of Dvl1 with Syt-1. We found that Wnt7a increases the interaction between endogenous Dvl1 and Syt-1 in cultured neurons (Fig. 7a,b). Conversely, blockade of endogenous Wnts with Sfrps decreases this binding (Fig. 7c,d). The effect of Sfrp is stronger than Wnt7a, consistent with the view that Sfrps block multiple Wnt proteins expressed by hippocampal neurons^39^,^40^,^41^.

We next examined the impact of modulating Wnt activity on the localization of Dvl1 and Syt-1. Hippocampal cultures (14 and 21 DIV) were treated with Wnt7a and Sfrps, respectively, to maximize the impact of gain and loss of function of Wnt signalling. We estimated the quantitative level of colocalization between endogenous Syt-1 and Dvl1 using the Pearson's correlation coefficient (*r*; ref. 59) between Syt-1 and Dvl1. This quantitative measurement estimates the degree of overlap between fluorescence signals obtained in two channels such that an increase in colocalization of the two signals results in an increase in *r*. Consistent with our immunoprecipitation experiments, we observed that Wnt7a or Sfrps do not change the number of Dvl1 or Syt-1 puncta along axons (Supplementary Fig. 6a,b). However, Wnt7a significantly increases, whereas Sfrps decrease the Pearson's correlation between Syt-1 and Dvl1 (Fig. 7e,f). Taken together, these findings demonstrate that Wnts modulate the interaction between Dvl1 and Syt-1 at the presynaptic terminal.

### Dvl1–Syt-1 interaction is required for neurotransmitter release

**Figure 8:**
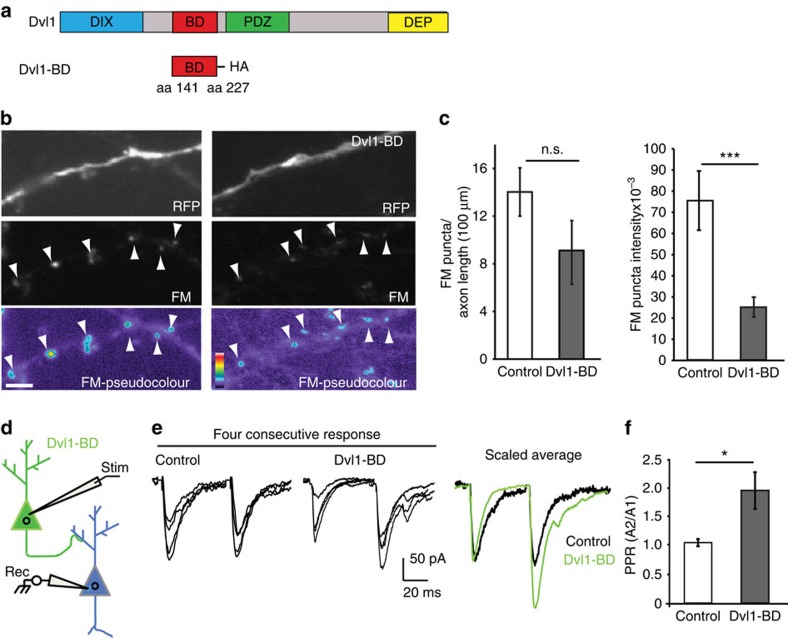
Binding between Dvl1 and Syt-1 is required for normal neurotransmitter release. (**a**) Schematic diagram showing the Dvl1 domain (BD) that binds to Syt-1. (**b**) FM-dye staining in low-density mature (21 DIV) hippocampal neurons expressing either RFP or RFP and Dvl1-BD in axons. Lower panel: pseudocolour images. Arrowheads indicating FM-dye-recycling sites. Scale bar, 5 μm. (**c**) Quantification reveals that expression of Dvl1-BD decreases the average intensity of recycling puncta without significantly affecting the number of FM-labelled sites along axons (*n*=18 control EGFP and 20 Dvl1-BD-transfected neurons from at least three independent experiments). ***P<0.001, unpaired Student's *t*-test. (**d**) Schematic representation of PPR experiment with stimulation of a transfected neuron (green) expressing EGFP or EGFP and Dvl1-BD and recording from a neighbouring untransfected neuron (blue). (**e**) Traces show four consecutive overlaid responses of representative cells, and the average response from these cells scaled to the first response. (**f**) Quantification shows the mean PPR (±s.e.m.) from all cells (*n*=10 pairs of control-stimulated cells from six independent cultures and *n*=9 pairs of Dvl1-BD-stimulated cells from five independent cultures). *P<0.05, unpaired Student's *t*-test.

A previous study showed that the region containing amino acids 141–227 of Dvl1 interacts with Syt-1 when overexpressed in cell lines^27^. We therefore reasoned that a peptide containing the binding domain (BD) of Dvl1 to Syt-1 could perturb the interaction between these two proteins, resulting in defective synaptic transmission. To test this, we expressed a construct coding for amino acids 141–227 of Dvl1 fused to HA (Dvl1-BD) together with full-length Dvl1-Flag and Syt-1-T7 in HEK-293 cells (Fig. 8a). As predicted, we observed that Dvl1-BD significantly decreases the binding between Dvl1 and Syt-1 when compared with control cells (Supplementary Figs 6c,d and 8).

To directly test the functional consequences of interfering with the interaction between Dvl1 and Syt-1, we investigated the effect of Dvl1-BD peptide on synaptic vesicle recycling in hippocampal neurons by determining the uptake of fixable FM1-43 on depolarization with high-potassium buffer (Fig. 8b). The number and intensity of FM-recycling sites were compared between axons expressing both Dvl1-BD and red fluorescence protein (RFP) and axons from control cells expressing only RFP (Fig. 8b,c). Although Dvl1-BD does not significantly affect the number of recycling puncta, a significant reduction in the fluorescence intensity of FM-recycling sites is observed (Fig. 8c). These results demonstrate that interference with the binding between Dvl1 and Syt-1 results in defective synaptic vesicle recycling.

To investigate the functional effect of the interaction between Dvl1 and Syt-1 on neurotransmitter release, we examined whether presynaptic expression of Dvl1-BD peptide affects PPR in hippocampal neurons. Enhanced green fluorescent protein (EGFP)-expressing control neurons or neurons expressing EGFP and Dvl1-BD were focally stimulated and the resulting EPSCs were recorded in postsynaptic untransfected neighbouring neurons (Fig. 8d). When Dvl1-BD-expressing cells were stimulated, we observed a significant increase in the PPR, corresponding to a decrease in *P*_r_ (Fig. 8e,f). Together, these results suggest that Wnt signalling modulates glutamatergic neurotransmitter release by regulating the direct interaction between Dvl1 and Syt-1.

## Discussion

Here we demonstrate that Wnt7a signalling modulates neurotransmitter release at the SC-CA1 synapse by targeting key components of the presynaptic release machinery. We report that, *in vivo, Wnt7a; Dvl1* deficiency results in impaired formation of the SNARE complex and a reduced number of synaptic vesicles close to the presynaptic membrane, with a corresponding reduction in the size of the RRP. Importantly, the Wnt-deficient mutant exhibits a decreased *P*_r_ at the SC-CA1 synapse. We also demonstrate that Wnt7a promotes the binding of the Wnt integrator Dvl1 with the calcium sensor Syt-1 and that this direct interaction is important for neurotransmitter release. Thus, Wnt7a signals to the release machinery to modulate neurotransmitter release by targeting the calcium sensor Syt-1.

Wnt signalling modulates neurotransmitter release at excitatory synapses. Few secreted and *trans*-synaptic proteins have been shown to modulate *P*_r_ (refs 18, 19, 60, 61). For example, acute exposure to BDNF specifically increases *P*_r_ at excitatory synapses^20^. Similarly, Wnt7a modulates neurotransmitter release at excitatory synapses, whereas inhibitory synapses remain unaffected. This effect of Wnt7a could be explained by the specific presence of Wnt7a receptors at excitatory synapses. Indeed, the Wnt co-receptor LRP6 is mainly present at excitatory synapses^62^. However, which receptors are involved in Wnt7a-mediated modulation of presynaptic function remains to be determined.

Previous studies have suggested that Wnts affect neurotransmitter release^25^,^26^. However, the *in vivo* role of Wnt signalling in this process and the mechanisms involved have not been elucidated. Here we demonstrate that *Wnt7a; Dvl1* KO mutant mice exhibit presynaptic defects at the SC-CA1 synapse, manifested by a decrease in input–output relationship, an increased PPR, a decrease in the rate of cumulative block of NMDA-R-mediated EPSCs by MK-801, a decreased ability to sustain transmitter release during trains of HFS and an activity-dependent reduction in quantal content. Consistently, acute blockade of endogenous Wnts with Sfrps increases PPR, without affecting synapse number in mature neurons. In contrast, gain of function of Wnt7a induces the converse effect. We observed that *Wnt7a; Dvl1* KO mutant mice exhibit a decrease in the number of vesicles proximal to the presynaptic plasma membrane, in the quantal content of evoked EPSCs and in the estimated quantal content of the RRP. As there is a strong correlation between *P*_r_ and the size of the RRP^5^,^19^,^30^,^49^,^50^,^51^, our data are consistent with a mechanism where changes in *P*_r_ are at least partly caused by a decrease in the number or availability of release-competent synaptic vesicles. These findings suggest a modulatory role for Wnt signalling in neurotransmitter release. Two findings support this notion. First, the decrease in quantal content in the *Wnt7a; Dvl1* KO mice is more pronounced at higher frequency. Second, blockade of Wnts abolishes the HFS-induced short-term change in *P*_r_. Together, these findings suggest a role for Wnts in adapting presynaptic responses to changes in neural activity.

How does Wnt signalling modulate neurotransmitter release? A possible target for this regulation is Syt-1, a well-established Ca^2+^ sensor that plays a crucial role in vesicle exocytosis^2^,^10^,^11^,^12^,^13^,^14^. Syt-1 promotes neurotransmitter release by forming a complex with the SNARE proteins Stx-1, SNAP25 and VAMP2. On Ca^2+^ entry, following the arrival of an AP at the presynaptic terminal, the SNARE complex promotes rapid vesicle fusion and release of neurotransmitter^2^,^6^,^7^,^8^. Here we demonstrate that Dvl1 directly interacts with Syt-1 at synapses, whereas its interaction with the t-SNARE proteins Stx-1 and SNAP25 is indirect. On the basis of these findings, we suggest that the interaction between Dvl1 with Syt-1 promotes the formation and/or stabilization of a complex containing Syt-1, Stx-1 and SNAP25. This process could increase the availability of SVs for release at excitatory synapses in a Wnt-dependent manner. Four findings are consistent with this hypothesis. First, we found that the *Wnt7a; Dvl1* mutant exhibits defects in the formation of the SNARE complex. Second, these mice have fewer vesicles proximal to the plasma membrane. Third, the size of the RRP and number of vesicles available for release are significantly reduced when Wnt signalling is compromised. Fourth, Wnt7a promotes, whereas Wnt antagonists reduce the interaction between Dvl1 and Syt-1. Importantly, blockade of this interaction in the presynaptic neuron using a specific Dvl1 peptide significantly decreases neurotransmitter *P*_r_ similarly to the effect observed in the Syt-1 KO mouse^63^.

In summary, we propose that Wnts are tonic/modulatory signals that regulate *P*_r_ by targeting Syt-1, a key component of the release machinery. The regulatory interaction between Dvl1–Syt-1 provides a direct mechanism for dynamic modulation of neurotransmission by extracellular signals allowing synapses to adapt to different demands.

## Methods

### Neuronal and cell cultures

Hippocampal cultures were prepared according to ref. 64. Briefly, primary hippocampal cultures were isolated from Sprague–Dawley rat embryos (embryonic day 18) obtained from the UCL animal facility and cultured in N2/B27 medium for 14 or 21 DIV. Mature neurons (younger: 14 DIV or older: 21 DIV) were treated with Wnt7a (R&D) or Sfrps (R&D), respectively, as Wnt7a acts strongly on younger cultures where endogenous Wnt levels are lower, whereas Sfrps are more effective in older cultures where higher levels of endogenous Wnts could be blocked. High- or low-density hippocampal neurons (250 and 50–100 cells mm^−2^, respectively) were treated with a combination of recombinant Sfrp1 (2.5 mg ml^−1^), Sfrp2 (200 ng ml^−1^) and Sfrp3 (250 ng ml^−1^) or with Wnt7a (100 ng ml^−1^; R&D Systems) either acutely (30 min or 3 h) or for a long time period (16 h), as outlined in the text. For recycling experiments, neurons were transfected using Amaxa nucleofection (Lonza) before plating. EGFP-actin (kindly provided by Dr Y. Goda), RFP (from Clontech) and a Dvl1 construct consisting of the region between amino acids 141 and 227 fused to haemagglutinin tag (Dvl1-BD, generated in our laboratory) were used. HEK-293 cells were transfected with Dvl1-Flag, Syt-1-T7 and Dvl-BD-HA using Fugene (Promega). Transfection efficiency for each experiment was tested by immunofluorescence and only experiments showing good transfection levels were used.

### Immunofluorescence and image acquisition analyses

Dissociated neurons were fixed with ice-cold 100% methanol, permeabilized with 0.025% Triton, blocked with 5% BSA and then incubated with primary antibodies overnight at 4 °C. Primary antibodies against vGlut1 (Chemicon 1:5,000 from the company stock), PSD-95 (Thermo Scientific: 1.6 ng μl^−1^), Tuj1 (Abcam; 0.8 ng μl^−1^), Dvl1 (SIGMA; 3 ng μl^−1^), GluN1 (Synaptic Systems; 2 ng μl^−1^), Syt-1 (Synaptic Systems; 1:1,000 from company stock) and VAMP2 (Synaptic Systems; 1 ng μl^−1^) were used. Secondary antibodies conjugated to Alexa 488, Alexa 568 and Alexa 647 were from Molecular Probes. Fluorescence images of neurons with a typical pyramidal morphology were captured with a Leica TCS SP1 confocal microscope using a × 63 oil objective (numerical aperture=1.32) and a z-step of 0.2 μm, producing image stacks of 157.8 × 157.8 μm with an average *z* depth of ∼5 μm. Images were acquired with Leica software and were analysed with Volocity software (PerkinElmer). The number of pre- and postsynaptic puncta, their colocalization and Tuj1 volume were detected using custom Volocity protocols using standard thresholding techniques. Punctum density was normalized to Tuj1 volume. Pearson's correlation coefficients were calculated using Volocity software from compressed confocal *z*-stacks. Analyses of colocalization were performed from images of isolated axons stained for Syt-1 and Dvl1. For these experiments, hippocampal cultures of different ages (14 and 21 DIV) were used to maximize the effect of Wnt7a and Sfrps, respectively, as Wnt7a has a stronger effect in younger cells (14 DIV), whereas Sfrps are more effective in older cultures.

### Mutant mice

All animal procedures were performed in accordance with the Animals Scientific procedures Act UK (1986) following approval from the UCL ethical committee and the Home Office. C57Bl/6 J *Dvl1* mutant mice were obtained from heterozygous crosses, whereas *Wnt7a; Dvl1* KO mice were obtained from crosses of *Wnt7a*^*−/+*^ with *Dvl1 KO* mice. Control mice were age-matched (P21) WT C57BL/6 animals (from Charles River). Genotypes were determined by three-primer PCR using DNA samples from ear clipping as previously described^29^. P21 animals were used in all experiments.

### Protein preparations and immunoprecipitation

Post-nuclear fractions from brain lysates were obtained from adult WT or *Wnt7a*^*−/−*^*; Dvl1*^*−/−*^ double-mutant (*Wnt7a; Dvl1* KO) mice. Briefly, hippocampi from each genotype were quickly isolated and triturated in 1 ml of SNARE buffer (50 mM HEPES; 2 mM MgCl_2_, Sucrose 250 mM). After centrifugation, samples were quantified and supplied with 1% w/v SDS and were loaded on a two-part (8–15%) step gel. To confirm that the complex observed was sensitive to boiling, an identical sample was boiled for 5 min and analysed at the same time. Antibody against Syntaxin-1 (Synaptic System; 1:1,000 from company stock) was used to identify the SNARE complex according to ref. 57. To evaluate the amount of the SNARE complex in WT or *Wnt7a; Dvl1* KO mice, the ratio between total Stx-1 and Stx-1 in the SNARE complex was quantified after the samples were boiled for 5 min. For synaptosomal preparation, adult mouse brains were homogenized in buffer A (0.32 M sucrose, 4 mM HEPES, pH 7.4, plus protease and phosphatase inhibitors) and centrifuged at 800*g* for 10 min. All procedures were performed at 4 °C. The supernatant was clarified by centrifugation at 9,000*g* for 15 min, and the pellet containing myelin and synaptosomal and mitochondrial structures was resuspended in buffer A and layered on top of a discontinuous gradient containing 0.8/1.0/1.2 M sucrose in 4 mM Hepes, pH 7.4, plus protease inhibitors, and were centrifuged at 82,500*g* for 90 min. Synaptic membranes were taken from a 1–1.2-M interface and resuspended in buffer B (0.32 M sucrose, 4 mM Hepes, pH 7.4 and 150 mM NaCl plus inhibitors). Proteins were quantified by Lowry assay. For immunoprecipitation experiments, mature high-density hippocampal cultures were lysed in Triton buffer (50 mM Hepes-NaOH, 0.1 M NaCl, 4 mM EGTA, 2 mM MgCl_2_, 0.5% Triton X-100). To examine the colocalization between Dvl1 and Syt-1, neurons from 14 or 21 DIV cultures were used to maximize the effect of Wnt7a and Sfrps, respectively. Cell lysates were incubated with 3 μg of Dvl1 monoclonal antibody (Santa Cruz; stock 200 ng μl^−1^) and protein G–Sepharose beads (Invitrogen) overnight at 4 °C. Samples were analysed using western blot analyses. Antibodies against Syt-1, Sxt-1, SNAP25 and VAMP2 were from Synaptic Systems (all antibodies were used at 1:1,000 dilution from company stock). Dvl1 polyclonal antibody was obtained in our laboratory^65^. For immunoprecipitation experiments in HEK-293 cells, T7 monoclonal antibody (Novagene), Flag polyclonal antibody (Sigma) and T7 polyclonal antibody (Millipore) were used (all antibodies were used at 1:1,000 dilution from company stock). Membranes were probed with horseradish peroxidase-coupled anti-rabbit or anti-mouse antibodies and developed using ECL reagent (Amersham used at a dilution 1:2,000 from company stock). Quantification of band intensity was performed using ImageJ software (National Institutes of Health).

### Yeast two-hybrid assays

The full-length coding regions of Dvl1, Syt-1, Sxt-1A, Vamp2 and SNAP25 were fused in-frame to the GAL4 DNA-BD of pGBKT7 (BD) or to the GAL4 activation domain (AD) of pGADT7 (Clontech). Primers for Dvl1 were forward (Fwd): 5′-CGTACAGAATTCGCGGAGACCAAAATCAT-3′ and reverse (Rvs): 5′-GGCTAGTCGACCATGATGTCCACAAAG-3′. Primers used for Syt-1 were Fwd: 5′-AAGTACGAATTCGTGAGTGCCAGTCGTCCTGAGG-3′ and Rvs: 5′-AAGTACCTCGAGCTTCTTGACAGCCAGCATGG-3′. For Sxt-1A Fwd: 5′-AAGTACATCGATACAAGGACCGAACCGAGGAGC-3′ and Rvs: 5′-AAGTACCTCGAGTCCAAAGATGCCCCCGATGG-3′. Primers used for VAMP2 were Fwd: 5′-AAGTACGAATTCTCGGCTACCGCTGCCACCG-3′ and Rvs: 5′-AAGTACCTCGAGAGTGCGGCTGGGGGTGGG-3′ and for SNAP25 Fwd: 5′-AAGTACGAATTCGCCGAAGACGCAGACATGCGC-3′ and Rvs: 5′-AAGTACCTCGAGACCACTTCCCAGCATC-3′. Empty pGBKT7- and GAL4 DNA-BD Dvl1 fusions were transformed into the yeast strain AH109 (MATa), whereas the empty pGADT7 and GAL4 AD fusions were transformed into the Y187 strain (MATα from Clontech). Three yeast transformants for each plasmid combination were mated on rich YPDA medium and selected under nutrition restriction on plates containing synthetic media without Leucine and Tryptophan (Clontech). Protein interactions were then assayed by monitoring growth on synthetic media lacking Histidine or Histidine and Adenine (Clontech).

### Electron microscopy

For ultrastructural analyses, mature hippocampal neurons (21 DIV) were fixed with 2% paraformaldehyde (PFA), 1.5% glutaraldehyde in 0.1 M sodium cacodylate buffer (pH 7.3) for 40 min at room temperature. Coverslips were post fixed in 1% w/v OsO_4_ in Cacodylate buffer for 1 h, stained in aqueous uranyl acetate for 45 min, dehydrated in graded alcohol and embedded in resin. For *in vivo* analyses, brains of P21 WT (*n*=3) and *Wnt7a; Dvl1* KO (*n*=3) were dissected and immersion-fixed with 4% w/v PFA, 0.5% v/v glutaraldehyde in 0.1 M Millonig's phosphate buffer (pH 7.4) overnight at 4 °C. Samples were rinsed in 0.1 M Millonig's buffer (0.1 M NaH_2_PO_4_ and 0.1 M Na_2_HPO_4_) and coronal sections of 200 μm were obtained using a vibroslicer (Campden Instruments—MA752). Ultrathin sections of ∼70 nm of silver–gold interference colour were cut and collected on a 200-mesh grid. Photographs were taken at × 40,000, with a JEOL 1010 microscope. Samples were only used if ultrastructural analyses comply with the parameters of a good electron micrograph (that is, round mitochondria and defined synaptic structures). Digital analyses were performed blind to experimental condition using ImageJ software (National Institutes of Health). Only non-perforated asymmetric synapses were considered. The postsynaptic density (PSD) length and number of vesicles were measured manually. Vesicles were measured either within 50 nm from the plasma membrane and were defined as proximal to the plasma membrane or within a region spanning from 50 to 150 nm from the plasma membrane. The number of vesicles was normalized to 100 nm of PSD length. We chose the PSD length for normalization as its borders can be easily identified with accuracy.

### Synaptic vesicle recycling

Twenty-one DIV hippocampal neurons previously transfected with RFP or with RFP together with Dvl1-BD were incubated for 5 min at 37 °C in warm Krebs–Ringer solution (125 mM NaCl, 25 mM HEPES, pH 7.4, 5 mM KCl, 2 mM CaCl_2_, 1.2 mM MgSO_4_, 1.2 mM KH_2_PO_4_ and 6 mM glucose) supplemented where indicated with 110 mM KCl (substituted for NaCl) and 10 μM APV and CNQX and containing 10 μM of fixable FM1-43 (Invitrogen). After incubation, cultures were washed three times in warm Krebs–Ringer buffer, and then fixed and processed for immunofluorescence. Images were captured with a BX60 Olympus microscope using a × 100 oil immersion objective and a charge-coupled device camera (Orca ER; Hamamatsu). Axons were traced manually, and the number and intensity of FM1-43 puncta were quantified using MetaMorph (Molecular Devices).

### Electrophysiology

Cultured hippocampal neurons or acute hippocampal slices (300 μm) were placed in a chamber on an upright microscope and continuously perfused at room temperature with recording solution containing (in mM): NaCl (125), NaHCO_3_ (25), KCl (2.5), NaHPO_4_ (1.25), CaCl_2_ (1), MgCl_2_ (1) and D-glucose (25) and bubbled with 95% O_2_/5% CO_2_. Cells were patched in the whole-cell voltage-clamp configuration using microelectrodes (resistance 5–8 MΩ) pulled from borosilicate glass (Harvard GC150F-7.5) and filled with pipette solution containing (in mM): D-gluconic acid lactone (139), HEPES (10), EGTA (10), NaCl (10), CaCl_2_ (0.5), MgCl_2_ (1), ATP (1) and GTP (0.5) adjusted to pH 7.2 with CsOH. When recording miniature currents, 100 nM tetrodotoxin (TTX) (Abcam PLC) was included in the recording solution. Miniature or evoked EPSCs were recorded at −60 mV in the presence of 10 μM bicuculline (Tocris Bioscience), whereas IPSCs were recorded at 0 mV in the presence of 10 μM 6-cyano-7-nitroquinoxaline-2,3-dione (CNQX) (Tocris Bioscience) and 50 μM APV (Tocris Bioscience). Evoked postsynaptic currents were elicited using a bipolar concentric electrode (FHC) attached to a Grass S48 stimulator to depolarize axons close to the patched cell. In hippocampal slices, the stimulating electrode was placed in the stratum radiatum ∼100–200 μm from the whole-cell patched neuron in the CA1 layer. In hippocampal cultures, large cells with a pyramidal morphology were patched and the stimulating electrode was placed within ∼100–200 μm of the patched cell. Paired-pulse (PP) stimuli were delivered at a rate of 0.2 Hz with an interstimulus interval of 50 ms for EPSCs, and an interval of 100 ms for IPSCs. The PPR was calculated as the ratio of the peak amplitude of the second response over the first response. For PP and RRP recordings, the stimulus intensity was altered from cell to cell to give the minimum reproducible response. For input–output analyses, stimuli were delivered at 3, 6, 9, 12 and 15 V at a rate of 0.1 Hz and at least three responses were averaged per stimulation intensity per cell. QX-314 (Insight Biotechnology; 10 mM) was used in the pipette solution to block AP firing in the patched cell. MK-801 experiments were performed on acute slices of WT and *Wnt7a; Dvl1* KO in the absence of Mg^2+^. The recording solution remained identical to the whole-cell recording, but contained CaCl_2_ (2.7 mM) and no MgCl_2._ NMDA-R-currents were evoked by 80 stimulations at 0.1 Hz in presence of MK-801 (Sigma-Aldrich; 30 μM), CNQX (10 μM) and Bicuculline (10 μM). Currents were normalized to the first NMDA-EPSC in the presence of MK-801. An exponential was fit to the normalized peak current against stimulus number to estimate the decay time constant.

For unitary PPR recordings in cultured neurons, we evoked postsynaptic currents using a bipolar theta glass electrode (filled with recording solution) attached to a Grass S48 stimulator. Nimodipine (Tocris Bioscience; 1 μM) was also introduced in the pipette solution to block postsynaptic L-type calcium channels and QX-314 (Insight Biotechnology; 10 mM) was used in the pipette solution to block AP firing. The recording solution remained identical to the whole-cell recording solution, but contained CaCl_2_ (2 mM) and MgCl_2_ (1 mM). The stimulating electrode was applied on the surface of an identified EGFP-expressing neuron, which was co-transfected with PSC2 used as an empty vector or the Dvl1-BD. Ten to one hundred responses were averaged for each cell, and the monosynaptic (response latency <4 ms) peak amplitude was measured.

For HFS PPR analyses in hippocampal slices, recording microelectrodes were positioned in the stratum radiatum of the CA1 region, while concentric bipolar stimulating electrodes were placed in SC afferent fibres to record field EPSPs. A stimulation protocol involving a PP 50 ms apart was delivered every 10 s with a stimulation strength set to ∼50% of the maximal response. HFS consists of a single train of 100 stimuli at 100 Hz following a 10–15-min baseline recording. Sfrps were applied 10 min before HFS.

For RRP estimation, CA1 cell EPSCs were recorded in response to 2.5–3-s duration trains of local electrical stimulation of the SC at 20 Hz. Quantal content was estimated from the ratio of EPSC peak amplitude to mEPSC amplitude and from the integrated EPSC charge passed during the period between the end of one EPSC stimulus artefact and the beginning of the next EPSC stimulus, divided by the integral of the average mEPSC (WT: 118±16.2 fC; *Wnt7a; Dvl1* KO: 115±18.3 fC). Stimulus artefacts were digitally removed for clarity. EPSCs were corrected for spill-over by using the current level immediately preceding each stimulus artefact as the reference current for measurement of the succeeding EPSC. For sucrose experiments, RRP size in cultured neurons was determined by hypertonic solution in the presence of 100 nM TTX and 10 μM Bicuculline. The recording solution remained identical to the whole-cell recording solution, but contained CaCl_2_ (2 mM) and MgCl_2_ (2 mM). The hypertonic solution was obtained by adding sucrose (500 nM) to the extracellular solution. This solution evoked a large initial transient current that declined to a low steady-state level over ∼3–4 s; hypertonic solution was applied for 10 s to deplete the RRP fully. The size of the RRP was calculated by integrating the evoked current, corrected by subtracting the amount of steady-state exocytosis that occurred during the hypertonic solution flow.

All currents were recorded using an Axopatch 200B amplifier, filtered at 1 kHz and digitized at 10 kHz using WinEDR software. Currents were analysed using a combination of WinEDR and WinWCP (freely available at http://spider.science.strath.ac.uk/sipbs/ software_ses.htm).

### Estimation of RRP size

RRP size was estimated using cumulative charge^54^,^55^ for both sucrose-evoked transmitter release at synapses in cultured hippocampal neurones and in analysis of 20-Hz stimulus-evoked trains of EPSCs in hippocampal slices. The RRP estimated from the cumulative charge during an EPSC train was corrected for vesicle recycling as detailed in the appendix of ref. 55. This method uses two independent equations to estimate the vesicle fusion efficiency (*f*e), which are solved numerically to estimate *f*e and *α*, the rate constant for vesicle recycling:


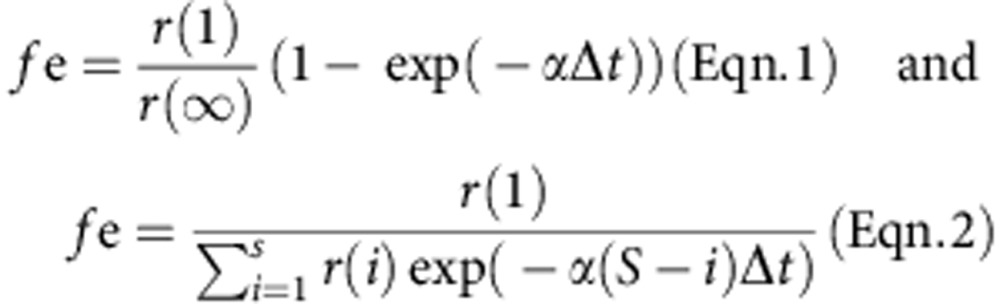


*r*(1) is the charge of the first EPSC in the train, *r*(i) the charge passed by the *i*th EPSC, *r*(∞) was calculated from the average charge of the last 10 EPSCs in the train and Δ*t* is the stimulus interval in the train (50 ms). The RRP was estimated as RRP=*r*(1)/*f*e.

### Statistical analysis

Values given are mean±s.e.m. Data presented correspond to at least three independent experiments, if not otherwise stated. When possible, the experiments were performed blind, and data were analysed blind to the experimental condition. For all data sets, sample normality and homogeneity were tested by Lillieford and *χ*^2^-test. For data sets with normal distribution, analysis of variance (ANOVA) or Student's *t*-tests were used. For western blot experiments including SNARE preparation two-way ANOVA with blocking was used to compensate for variability between experiments. For ultrastructural analysis data sets, which are non-normally distributed, the Kruskal–Wallis test was used. For the mean mEPSC and mIPSC frequency, which are also non-normally distributed, the Mann–Whitney test was used.

## Additional information

**How to cite this article:** Ciani, L. *et al*. Wnt signalling tunes neurotransmitter release by directly targeting Synaptotagmin-1. *Nat. Commun.* 6:8302 doi: 10.1038/ncomms9302 (2015).

## Supplementary Material

Supplementary Information Supplementary Figures 1-8 and Supplementary References

## Figures and Tables

**Table 1:** Proteins of the release machinery that interact with Dvl1 in mature hippocampal neurons.

**Synaptic protein**	**Complex with Dvl1 (IP)**	**Direct interaction**
Syt-1	Yes	Yes
SNAP25	Yes	No
Stx-1	Yes	No
VAMP2	No	No
CASK	Yes	NE
Munc-18	No	NE
Complexin	No	NE

Dvl1, Dishevelled-1; IP, immunoprecipitate; NE, not examined.
