# Supplementary material for: Wnt signalling tunes neurotransmitter release by directly targeting Synaptotagmin-1
Source: Nat Commun. 2015 Sep 24;6:8302. doi: 10.1038/ncomms9302 (PMC4667432; doi:10.1038/ncomms9302)
Supplement: Supplementary Information — Supplementary Figures 1-8 and Supplementary References [file ncomms9302-s1.pdf]

SUPPLEMENTARY FIGURE LEGENDS

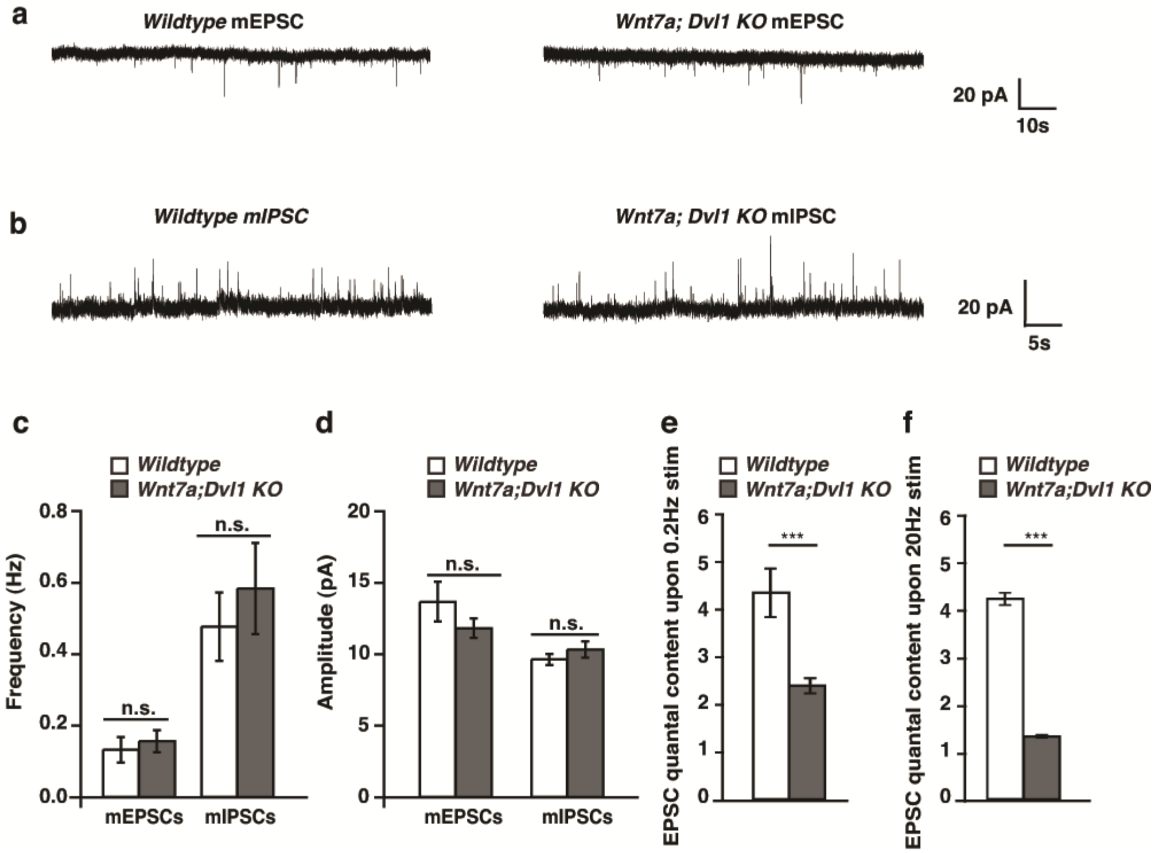

Supplementary Figure 1 Miniature synaptic transmission is unaffected in CA1 neurons of *Wnt7a; Dvl1* KO mice while quantal content is reduced (**a** and **b**) CA1 cells in acute hippocampal slices from P10-P14 *WT* or *Wnt7a; Dvl1* KO mice were whole cell patch-clamped and mEPSCs and mIPSCs were recorded. Example traces show no differences in mEPSC (**a**) or mIPSC (**b**) frequency or amplitude between *WT* and *Wnt7a; Dvl1* KO mice. (**c**) Quantification of mean mEPSC and mIPSC frequency. (**d**) Quantification of mean mEPSC and mIPSC amplitude (n=25 cells from 10 *WT* mice and 24 cells from 8 *Wnt7a; Dvl1* KO for mEPSC and n=23 cells from 11 *WT* mice and n=25 cells from 7 *Wnt7a; Dvl1* KO for mIPSC). n.s. = non-significant, unpaired Student's t-test for amplitude and Mann-Whitney test for frequency. (**e**) Quantification of quantal content for evoked EPSCs between *WT* and *Wnt7a; Dvl1* KO mice. Evoked synaptic currents were recorded by stimulation of SC axons while recording whole-cell currents in CA1 neurons. Quantal content was calculated by the direct method: EPSC/mEPSC

amplitudes (*WT*: 4.35 ± 0.51 quanta, n = 15; *Wnt7a; Dvl1* KO = 2.4 ± 0.16 quanta, n = 16). (**f**) Quantification of quantal content during 20Hz stimulation trains between *WT* and *Wnt7a; Dvl1* KO mice. (*WT*: 4.21 ± 0.13 quanta, n =12; *Wnt7a; Dvl1* KO = 1.32 ± 0.04 quanta, n = 14). P***< 0.001; unpaired Student's t-test.

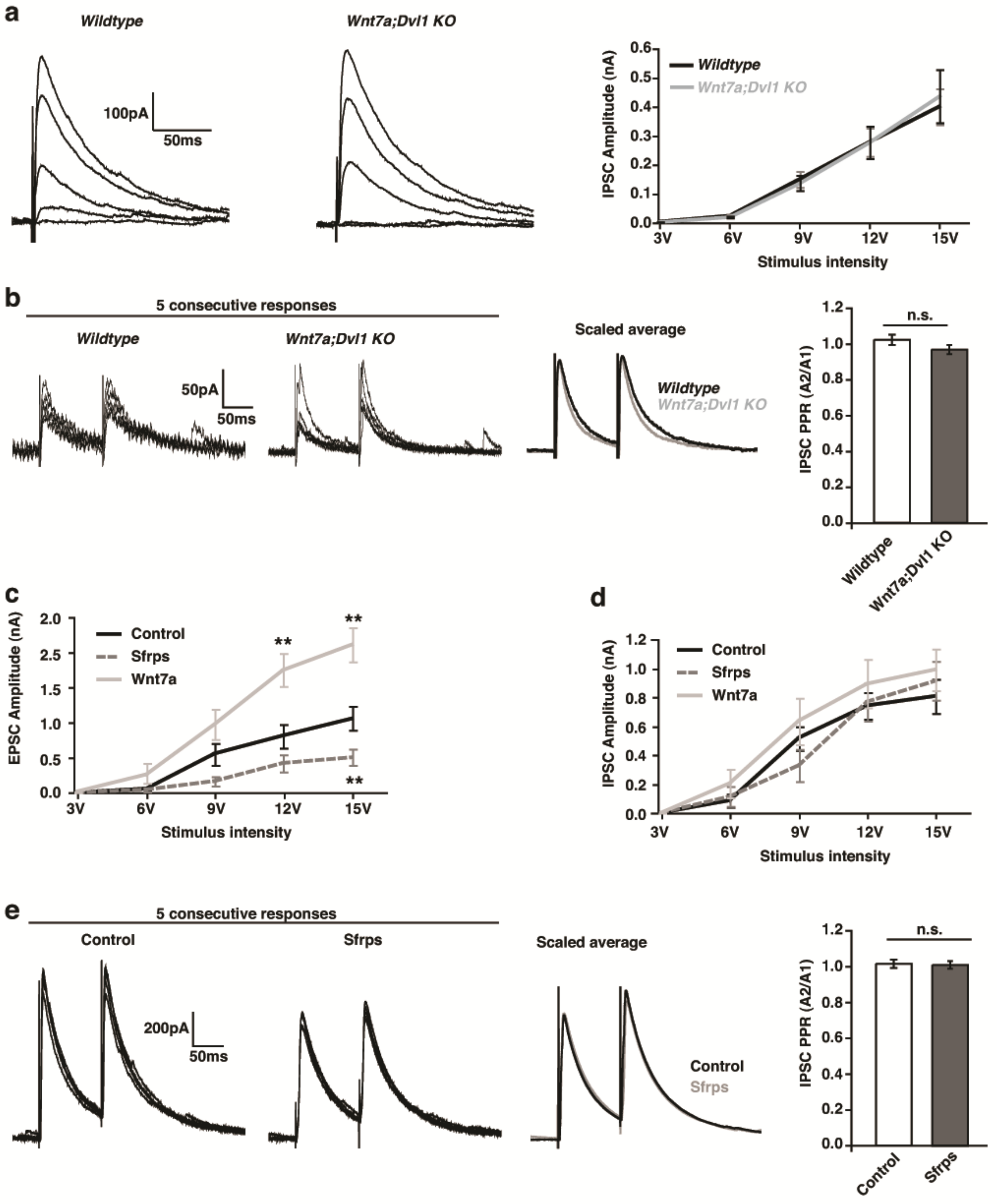

Supplementary Figure 2 Wnt signalling does not affect release probability at GABAergic synapses (**a**) Input-output relationship of evoked IPSCs at CA1 pyramidal cells in hippocampal slices from P21 *WT* and *Wnt7a; Dvl1* KO mice. Traces show responses of representative cells at increasing stimulus voltages (average of 3-5 responses for each stimulus strength). Graph shows mean IPSC amplitude (± s.e.m.) at each

stimulus voltage from all cells (n=12 cells from 4 animals for *WT*, 10 cells from 3 animals for *Wnt7a; Dvl1* KO). No difference is observed in the IPSC response at any of the stimulus intensities used. n.s. = non-significant, unpaired Student's t-test. (**b**) PPR of IPSCs in P21 *WT* and *Wnt7a; Dvl1* KO hippocampal slices. Traces show 5 consecutive overlaid responses of representative cells (left and middle panels), and the average response from these cells scaled to the first response (right panel). Graph shows mean PPR (± s.e.m.) from all cells (n=15 cells from 5 animals for *WT*, 17 cells from 7 animals for *Wnt7a; Dvl1* KO). No difference is observed in PPR between genotypes. (n.s. = non-significant, unpaired Student's t-test). (**c** and **d**) Input-output relationship of evoked EPSCs (**c**) and IPSCs (**d**) in mature hippocampal cultures treated with control, Wnt7a or a cocktail of Sfrps for 3 hours (n=17 cells from 8 cultures for control, 19 cells from 3 cultures for Wnt7a, 18 cells from 8 cultures for Sfrps). At higher stimulus intensities Wnt7a significantly increases, while Sfrps significantly decrease, EPSC amplitude. In contrast, no change in the IPSC response is observed at any of the stimulus intensities used. P**<0.01, n.s. = non-significant, one way ANOVA. (**e**) PPR of IPSCs in control and Sfrps treated mature hippocampal cultures. Traces show 5 consecutive overlaid responses of representative cells (left and middle panels), and the average response from these cells scaled to the first response (right panel). Graph shows mean PPR (± s.e.m.) from all cells (n=15 cells from 8 cultures for control, 18 cells from 7 cultures for Sfrps). No difference is observed in PPR following Sfrp treatment. n.s. = non-significant, unpaired Student's t-test.

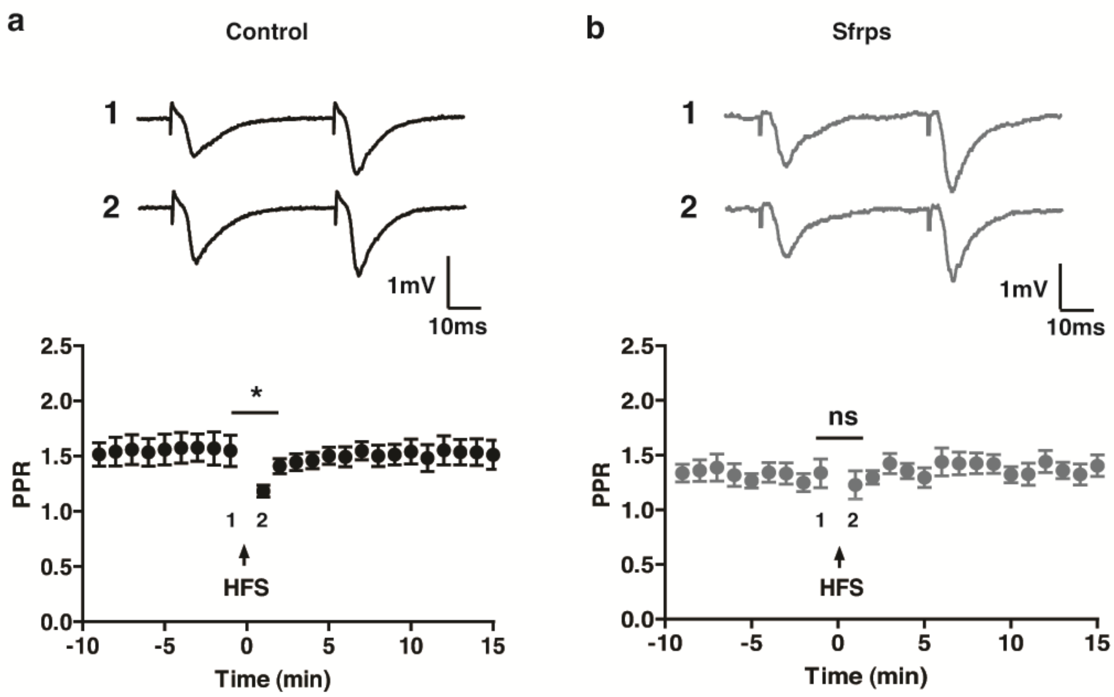

Supplementary Figure 3 Acute blockade of Wnts suppresses the increase in release probability induced by HFS (**a**) Top: Representative fEPSP traces show responses obtained 1 minute before (1) and 1 minute after HFS (2) in control. Bottom: Time course of paired-pulse recordings obtained from hippocampal slices before and after HFS (n=5 slices) P*<0.01, unpaired Student's t-test. (**b**) Top: Representative fEPSP traces show responses obtained 1 minute before (1) and 1 minute after HFS (2) in presence of Sfrps. Bottom: Time course of paired-pulse recordings obtained from hippocampal slices before and after HFS in presence of Sfrps. (n=7 slices). n.s. = non-significant, unpaired Student's t-test.

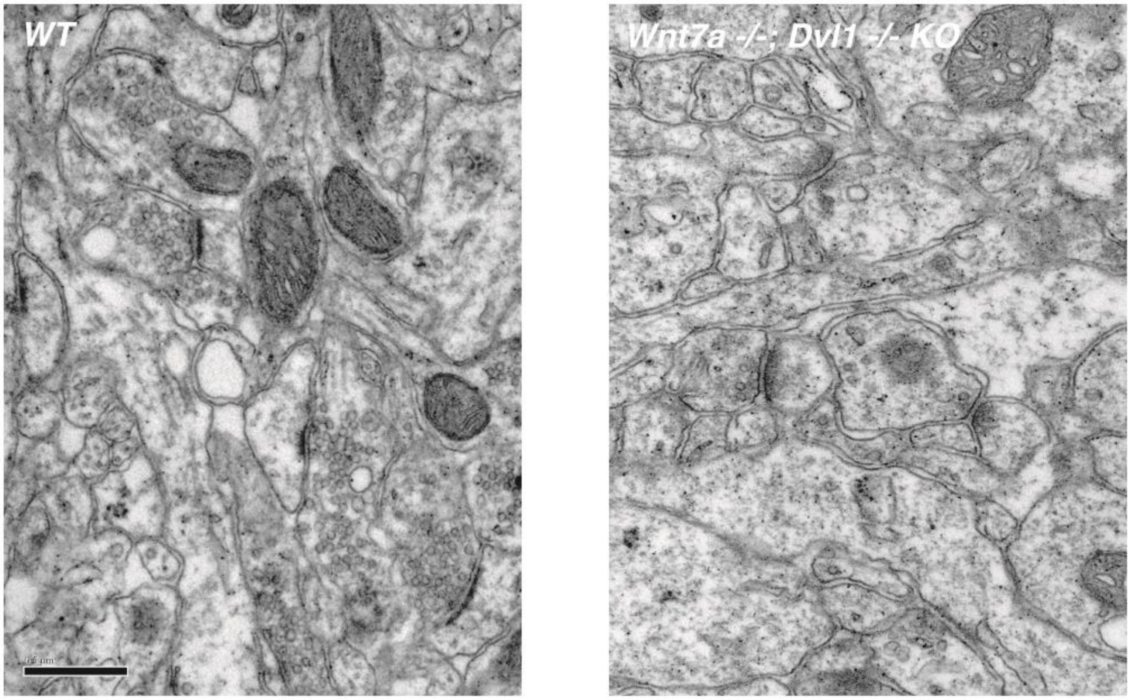

Supplementary Figure 4 Low magnification representative electron micrographs of the CA1 area of the hippocampus from *WT* and *Wnt7a; Dvl1* KO mice. Scale bar: 500 nm.

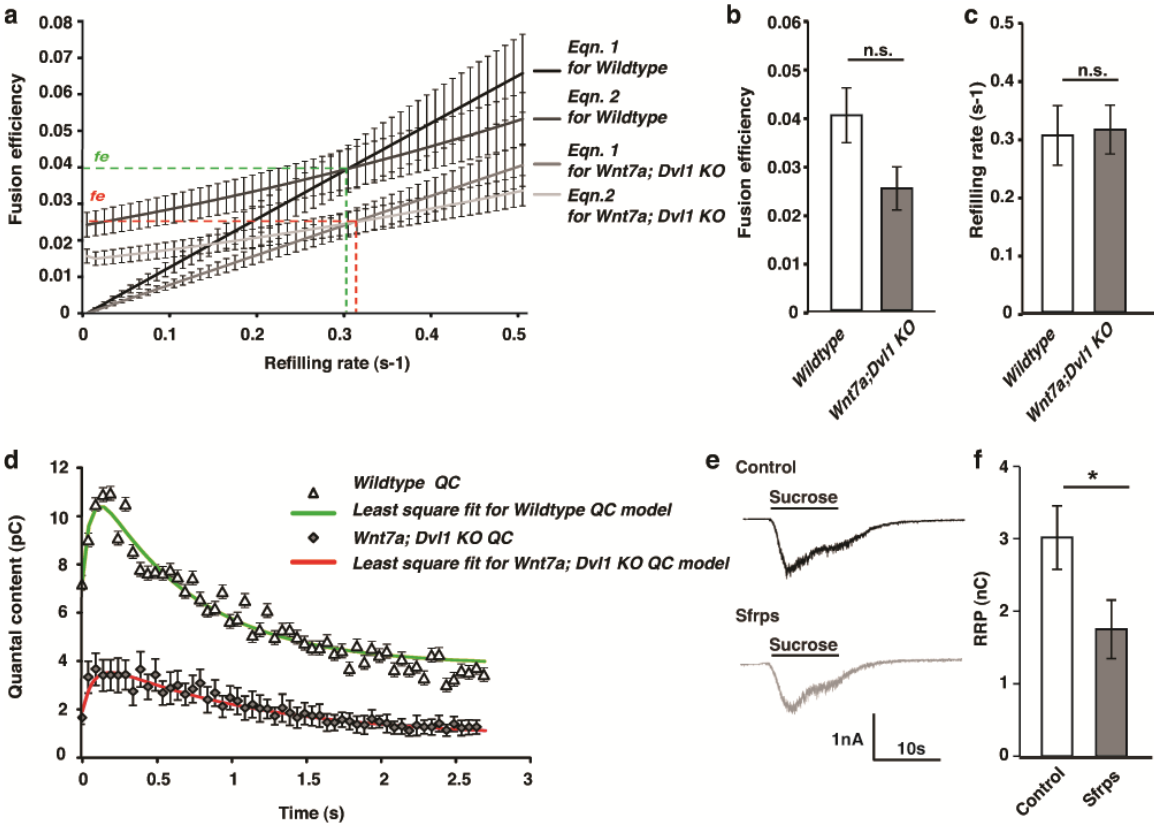

Supplementary Figure 5 Deficiency in Wnt signalling reduces release probability by affecting predominantly the RRP (**a**) The first order correction of the cumulative charge of trains of EPSCs (presented in Fig. 5) determines fusion efficiency and refilling rate as the intersection of these curves (dotted lines: green for *WT* and red for *Wnt7a; Dvl1* KO mice) as described in [1]. (**b** and **c**) No difference is seen in the fusion efficiency (**b**) or the refilling rate constant (**c**) between genotypes. n.s. = non-significant, unpaired Student's t-test. (**d**) Mean EPSC quantal content against time (s) during 20Hz trains of stimulation in *WT* and *Wnt7a; Dvl1* KO mice. Solid lines show least-squares fits of the release model to the data for *WT* (green line) and for *Wnt7a; Dvl1* KO mice (red line) respectively with parameters of RRP = 255 and 116 (46% decrease); $P_{R(rest)}$ = 0.027 and 0.016; $P_{R(ss)}$ = 0.047 and 0.035; τ_F = 59.8 and 81.6 ms and $k_{recycle}$ = 0.46 and 0.17 s^{-1}. Fitting the model to data from individual cells indicates the estimates for RRP (*WT*: 264 ± 63 (n = 12), *Wnt7a; Dvl1* KO: 105 ± 27 (n = 14) are significantly different (unpaired Student's t-test, P < 0.05) between *WT* and *Wnt7a; Dvl1* KO mice. The parameters estimated (WT and *Wnt7a; Dvl1* KO) are: $P_{R(rest)}$ = 0.024 ± 0.003 and 0.031 ± 0.012; $P_{R(ss)}$ = 0.048 ± 0.008 and 0.012 ± 0.068; t_F = 81 ± 19 and 162 ± 33 ms and $k_{recycle}$ = 0.27

± 0.09 and 0.31 ± 0.1 s^{-1}. (**e**) Representative responses evoked by hypertonic sucrose application from neurons exposed to control (black) or Sfrps (grey). (**f**) Sfrps decreases the RRP size in hippocampal mature neurons (n=17 cells for control, n=20 cells for Sfrps). P*< 0.05; unpaired Student's t-test.

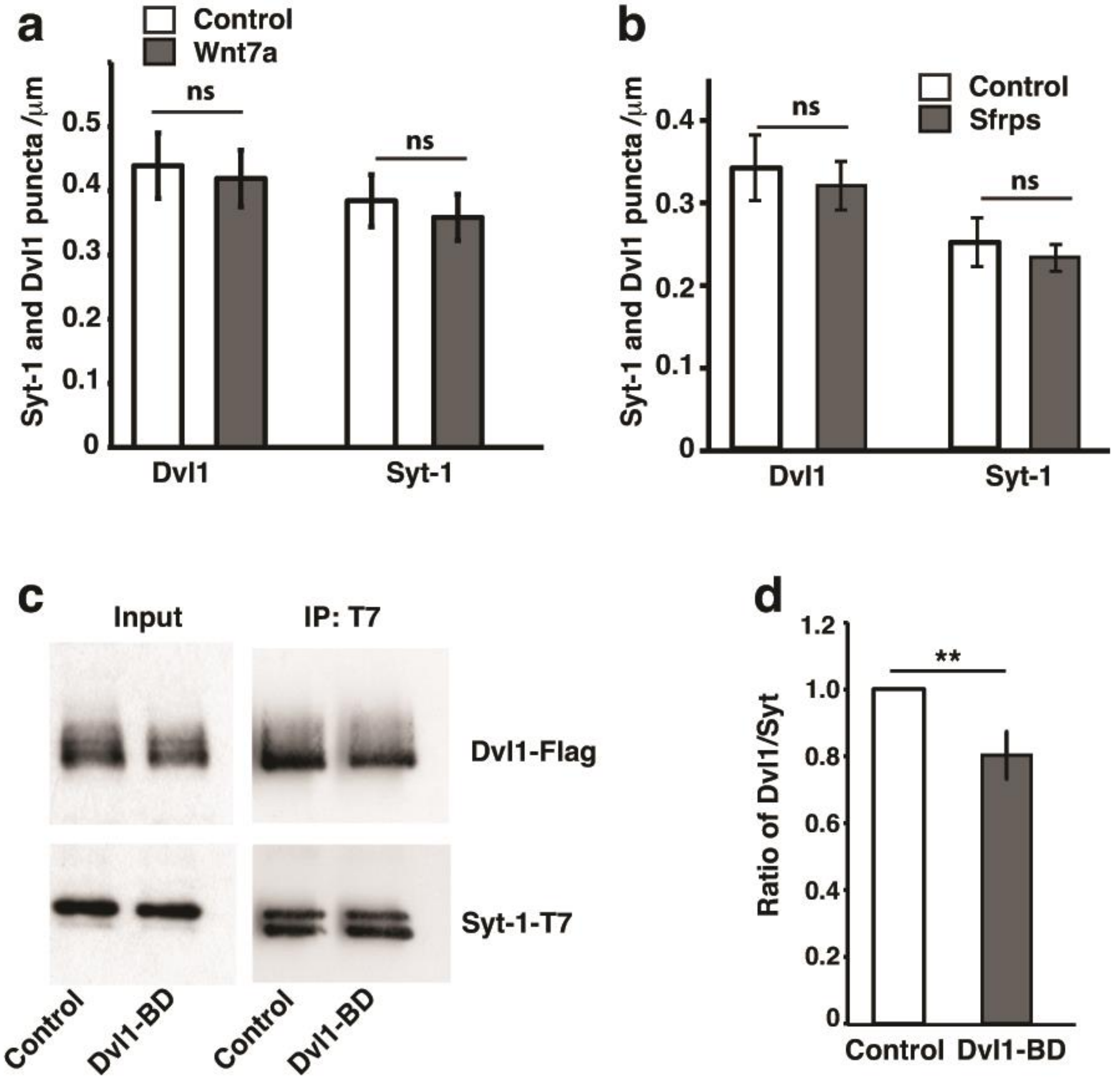

Supplementary Figure 6 The interaction between Dvl1 and Syt-1 is inhibited by Dvl-BD (**a**) Quantification shows that 30 min treatment with Wnt7a does not increase the number of Syt-1 or Dvl1 along the axons of hippocampal neurons (n=3 independent experiments). (**b**) Quantification shows that 30 min treatment with Sfrps does not increase the number of Syt-1 or Dvl1 along the axons of mature hippocampal neurons (n=3 independent experiments). (**c**) Representative immunoprecipitation from HEK 293 cells expressing Dvl1-Flag, Syt-1-T7 and Dvl1-BD-HA. Full-length blot provided in Supplementary Fig 8 (**d**) Quantification shows that expression of Dvl1-BD decreases the interaction between Dvl-1 and Syt-1 (n=6 independent experiments). $P^{**} < 0.01$, one-way ANOVA.

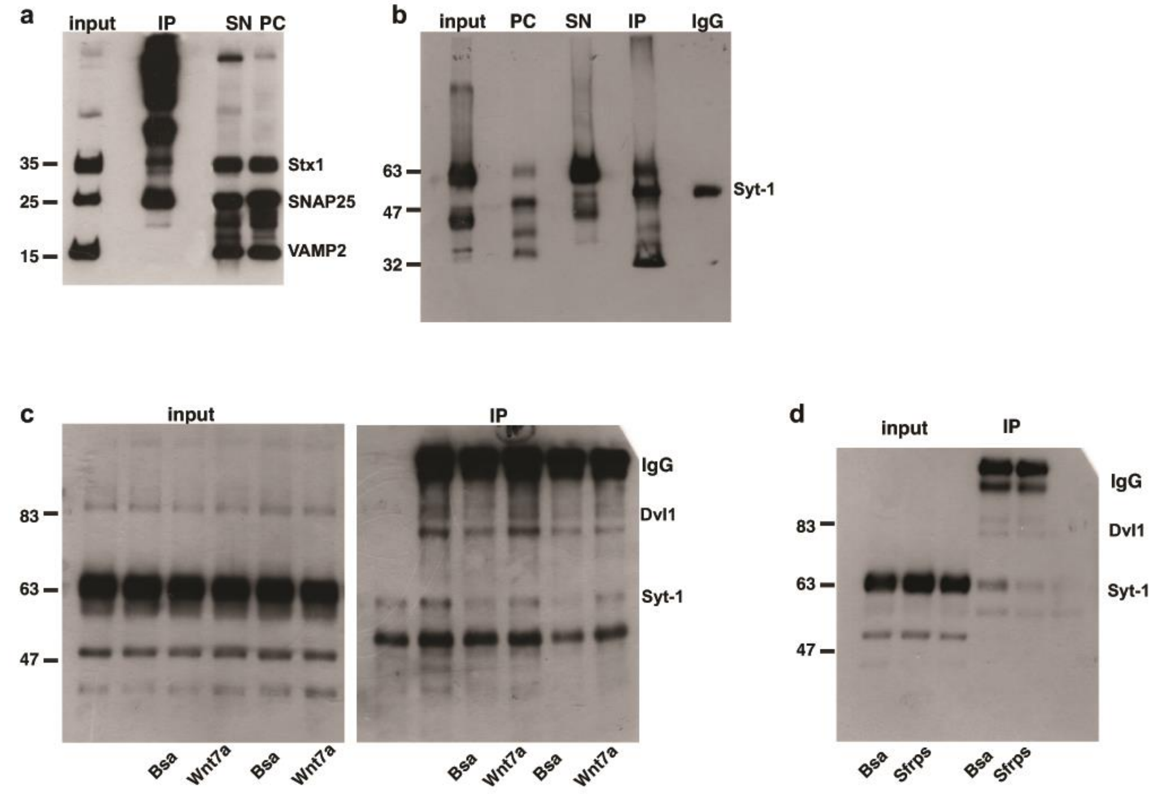

Supplementary Figure 7 Original gels from representative western blots shown in Fig 6 (a) Blot corresponding to panel (**c**) in Fig 6. **(b)** Blot corresponding to panel (**e**) in Fig 6. (**c**) Blot corresponding to panel (**a**) in Fig 7. (**d**) Blot corresponding to panel (**c**) in Fig 7.

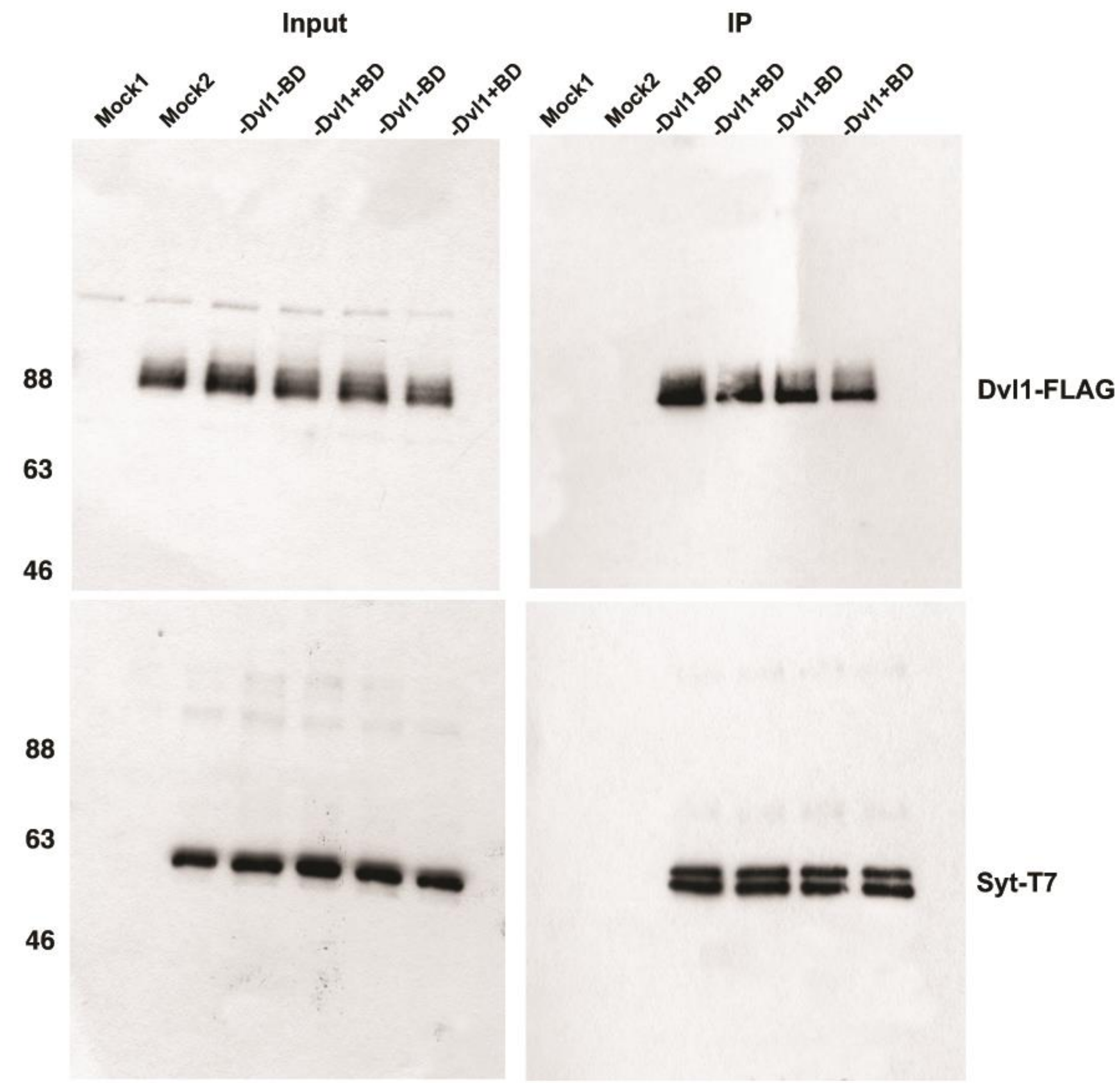

Supplementary Figure 8: Original gels from representative western blots shown in Supplementary Fig 6 panel **(c).**

SUPPLEMENTARY REFERENCES

1. Wesseling, J. F. & Lo, D. C. Limit on the role of activity in controlling the release-ready supply of synaptic vesicles. *J Neurosci* **22**, 9708-9720 (2002).
